# RNAi screening of uncharacterized genes identifies promising druggable targets in *Schistosoma japonicum*

**DOI:** 10.1371/journal.ppat.1013014

**Published:** 2025-03-28

**Authors:** Yuxiang Xie, Xiaoling Wang, Shaoyun Cheng, Wanling Liu, Cun Yi, Yanmin You, Wei Zhang, Yuepeng Wang, Enlu Tang, Jipeng Wang, Wei Hu

**Affiliations:** 1 State Key Laboratory of Genetic Engineering, Ministry of Education Key Laboratory of Contemporary Anthropology, School of Life Sciences, Fudan University, Shanghai, China; 2 Department of Infectious Diseases, Huashan Hospital, Fudan University, Shanghai, China; 3 College of Life Sciences, Inner Mongolia University, Hohhot, Inner Mongolia Autonomous Region, China; 4 National Institute of Parasitic Diseases, Chinese Center for Disease Control and Prevention (Chinese Center for Tropical Diseases Research), NHC Key Laboratory of Parasite and Vector Biology, WHO Collaborating Center for Tropical Diseases, National Center for International Research on Tropical Diseases, Shanghai, China; The University of Texas Health Science Center at Houston, UNITED STATES OF AMERICA

## Abstract

Schistosomiasis affects more than 250 million people worldwide and is one of the neglected tropical diseases. Currently, the treatment of schistosomiasis relies on a single drug-praziquantel-which has led to increasing pressure from drug resistance. Therefore, there is an urgent need to find new treatments. The development of genome sequencing has provided valuable information for understanding the biology of schistosomes. In the genome of *Schistosoma japonicum*, approximately 11% of the protein-coding sequences are uncharacterized genes (UGs) annotated as “hypothetical protein” or “protein of unknown function.” These poorly understood genes have been unjustifiably neglected, although some may be essential for the survival of the parasites and serve as potential drug targets. In this study, we systematically mined the highly expressed UGs in both genders of this parasite throughout key developmental stages in their mammalian host, using our previously published *S. japonicum* genome and RNA-seq data. By employing *in vitro* RNA interference (RNAi), we screened 126 UGs that lack homologs in *Homo sapiens* and identified 8 that are essential for the parasite vitality. We further investigated two UGs, *Sjc_0002003* and *Sjc_0009272*, which resulted in the most severe phenotypes. Fluorescence *in situ* hybridization demonstrated that both genes were expressed throughout the body without sex bias. Silencing either *Sjc_0002003* or *Sjc_0009272* reduced the cell proliferation in the body. Furthermore, *in vivo* RNAi indicated both genes are required for the growth and survival of the parasites in the mammalian host. For *Sjc_0002003*, we further characterize the underlying molecular cause of the observed phenotype. Through RNA-seq analysis and functional studies, we revealed that silencing *Sjc_0002003* reduces the expression of a series of intestinal genes, including *Sjc_0007312* (hypothetical protein), *Sjc_0008276* (vha-17), *Sjc_0002942* (PLA2G15), and *Sjc_0003646* (SJCHGC09134 protein), leading to gut dilation. Our work highlights the importance of UGs in schistosomes as promising targets for drug development in the treatment of the schistosomiasis.

## Author summary

Schistosomiasis, a parasitic disease affecting over 250 million people worldwide, is currently treated with a single drug, praziquantel, raising concerns about potential drug resistance. *S. japonicum*, the most pathogenic species, poses a significant health threat. Genome sequencing has revealed that approximately 11% of *S. japonicum* genes are uncharacterized, labeled as “hypothetical proteins” or “proteins of unknown function.” Despite their potential as therapeutic targets, these genes remain largely unexplored. In this study, we employed RNA interference (RNAi) to screen 126 highly expressed uncharacterized genes (UGs) throughout the key developmental stages from juvenile to adult *S. japonicum*. Silencing eight of these genes, including *Sjc_0002003* and *Sjc_0009272*, impaired parasite viability, reduced cell proliferation, and disrupted growth, survival, and reproduction in both *in vitro* and *in vivo* settings. RNA-seq analysis following the inhibition of *Sjc_0002003* identified critical downstream targets in the parasite’s gut, essential for its intestinal function. Our findings underscore the importance of uncharacterized genes as potential targets for new anti-schistosomiasis therapies, addressing the urgent need for alternative treatment options.

## Introduction

Schistosomiasis, a parasitic disease caused by blood flukes of the genus *Schistosoma*, affects over 250 million individuals globally[1]. The life cycle of schistosome is complex, involving a definitive human host and an intermediate snail host. In mammalian hosts, male and female worms mature and mate, with the adult female producing hundreds to thousands of eggs daily, which serve as a major pathological factor[2]. Praziquantel (PZQ) is currently the only first-line treatment for schistosomiasis, yet its widespread use raises concerns regarding the potential development of resistance in schistosomes [3]. Consequently, there is an urgent need to explore alternative therapeutic agents.

Identifying promising druggable targets is crucial for drug development. Recent large-scale RNA interference (RNAi) screening by Wang *et al*.[4] investigated over 2,000 genes in *S. mansoni*, revealing approximately 200 potential targets, many of which exhibit homology to human proteins. While this homology may facilitate drug development, it also poses a risk of adverse effects on human hosts. Thus, targeting schistosome proteins with no similarity to human counterparts may provide a safer therapeutic avenue.

The advent of genome sequencing has uncovered thousands of open reading frames encoding proteins, yet many of these remain functionally uncharacterized[5]. Uncharacterized genes (UGs) exhibit no detectable sequence or structural similarity to known proteins and are often overlooked in research despite their potential significance[6–9]. With advancements in sequencing technology, the genome of *S. japonicum* has been assembled to the chromosome level, with the latest version, *Sj*V3, containing 9,771 annotated genes[10]. Notably, 1,088 UGs, labeled as “hypothetical proteins” or “proteins of unknown function,” constitute approximately 11% of the genome and represent viable therapeutic targets due to their low or absent homologs in *Homo sapiens* (S1 and S2 Tables).

In this study, we selected UGs longer than 200 nucleotides with the top 100 expression levels in both sexes of *S. japonicum* during critical developmental stages (14-28 days post-infection)[11]. Following this, we conducted *in vitro* RNAi screening, identifying eight UGs essential for parasite survival. We further investigated the effects of UGs *Sjc_0002003* and *Sjc_0009272*, which produced severe phenotypes, on cell proliferation and the parasite growth, development and survival. For *Sjc_0002003*, which had the most pronounced effects, we demonstrated that its RNAi-mediated intestinal phenotype is linked to the downregulation of several intestinal genes, including *Sjc_0007312*, *vha-17*, *pla2g15*, and *Sjc_0003646*. Collectively, these findings underscore the importance of UGs in the survival of *S. japonicum* and their potential as promising therapeutic targets.

## Results

### *In vitro* RNAi screening identifies eight UGs essential for parasite vitality

**Fig 1:**
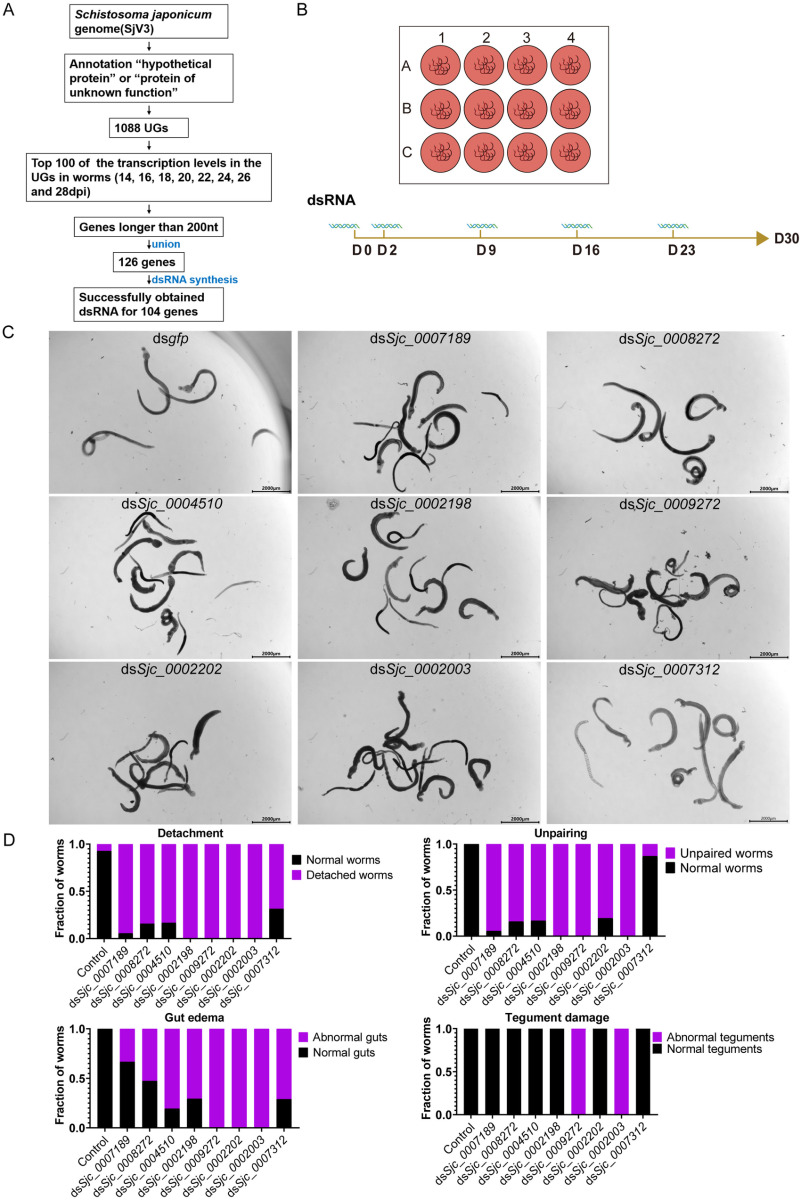
Eight UGs are essential for parasite vitality *in vitro.* (A) Pipeline for the identification of target UGs. (B) Schematic diagram depicting the RNAi screening strategy *in vitro*. (C) Worms from the control group (ds*gfp*) and the eight UGs (RNAi) group at day 30 of *in vitro* culture, observed under light microscopy. Control (RNAi) worms remain paired and attached to the bottom of the plate. Worms in the eight UGs (RNAi) group show decreased activity and have lost the ability to attach. **n** ≥ 15 worm pairs, three biological replicates. Scale bars: 2000 μm. (D) Plots depicting the relative fraction of animals showing normal or defective phenotypes after following 30 days RNAi treatments. **n** ≥ 40 worm pairs, four biological replicates.

To identify UGs in *S. japonicum*, we searched the annotation of the coding genes in its genome using the keywords “hypothetical protein” and “protein of unknown function” (Fig 1A). These 1,088 potential UGs accounted for approximately 11% of the entire genome’s coding genes (Fig 1A and S1 Table). After running *BLASTp* against the protein datasets of *Homo sapiens* and *Mus musculus* (https://www.ncbi.nlm.nih.gov), we identified 14 UGs with encoding proteins showing similarities to *Homo sapiens*, ranging from 16% to 66% (S2 Table). Additionally, we found 12 UGs with encoding proteins showing similarities to *Mus musculus*, ranging from approximately 21% to 75% (S3 Table). After filtering these genes, we focused on the remaining 1,071 UGs for further research.

To screen for essential UGs required for parasite survival, we first prioritized UGs with high transcription levels (TMM-normalized counts) in the parasite between 14-28 dpi (days post-infection) from our previous study[11], a key developmental window covering the transition from juvenile to adult stages in the mammalian host (Fig 1A and S4 Table). Assuming that highly expressed genes at each stage or sex may play vital roles in schistosomes, we selected the top 100 UGs in female and male worms at each stage based on our previously published RNA-seq data[11]. After consolidating these UGs and removing duplicates, we filtered out those with sequences shorter than 200 bp, resulting in a final dataset of 126 candidate targets (Fig 1A and S5 Table).

Among these 126 UGs, double-stranded RNA (dsRNA) targeting 104 UGs was successfully generated, while 22 UGs were not successful (S5 Table). For the RNAi screening, adult worms (30 dpi) were treated with dsRNA for 30 days *in vitro* (Fig 1B). The main phenotypic indicators included attachment, pairing, intestinal tract health, tegument condition, and vitality. Eight UGs were identified as essential for the survival of adult worms: *Sjc_0007189*, *Sjc_0008272*, *Sjc_0004510*, *Sjc_0002198*, *Sjc_0009272*, *Sjc_0002202*, *Sjc_0002003* and *Sjc_0007312* (Fig 1C and S6 Table). Among these, silencing *Sjc_0002003* and *Sjc_0009272* resulted in the most pronounced phenotypic changes, including the earliest appearance of phenotype and severe end-stage effects, prompting us to select them for further study (S6 Table and Fig 1C and 1D).

### *Sjc_0002003* and *Sjc_0009272* are expressed throughout juvenile and mature worms

**Fig 2:**
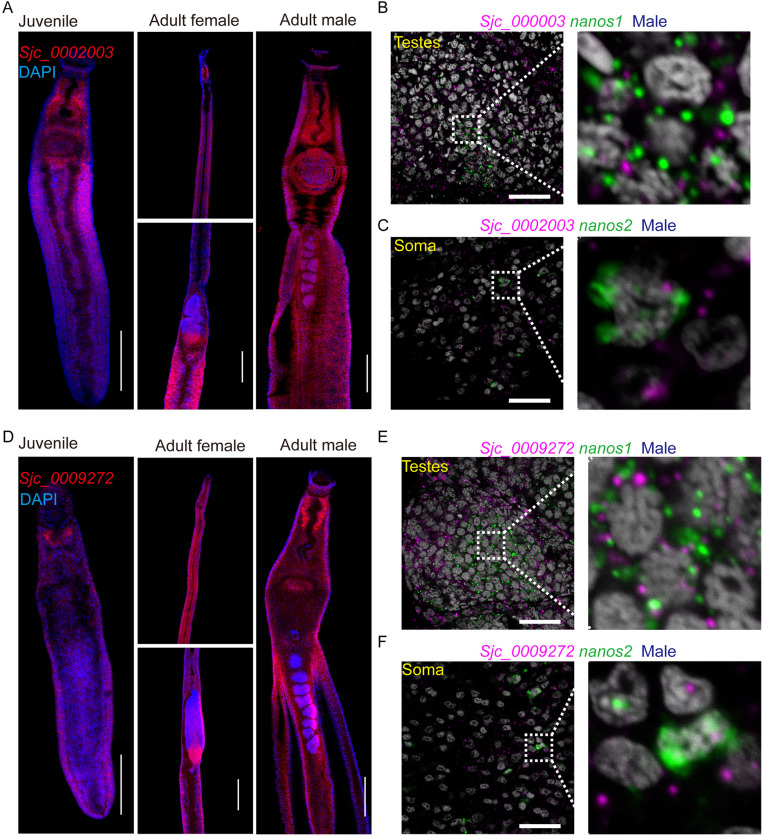
Localization of *Sjc_0002003* and *Sjc_0009272* mRNA in juvenile and mature worms. (A) Fluorescence *in situ* hybridization showing mRNA expression of *Sjc_0002003*. Juvenile worms (left), adult female (middle) and adult male (right). For adult females, two areas are highlighted: the head (top) and the middle body including ovary and vitellaria (bottom). Juvenile worms, 14 dpi. Adult worms, 26-30 dpi. **n** ≥ 18, three biological replicates. Scale bars: 200 μm. (B-C) Double FISH of *Sjc_0002003* with *nanos1* (B)or *nanos2* (C) in testes or soma of adult males. Right: magnified area in the white box on the left. Nuclei are shown in gray. **n** ≥ 8. Scale bars: 25 μm. (D) FISH showing mRNA expression of *Sjc_0009272*. **n** ≥ 18, three biological replicates. Scale bars: 200 μm. (E-F) Double FISH of *Sjc_0009272* with *nanos1* (E) or *nanos2* (F) in testes or soma of adult males. Right: magnified area in the white box on the left. Nuclei are shown in gray. **n** ≥ 8. Scale bars: 25 μm.

*Sjc_0002003* and *Sjc_0009272* were identified as highly expressed genes in *S. japonicum* from 14-28 dpi, according to our previous data[11]. We then analyzed their tissue specificity in juvenile and mature worms. Fluorescence *in situ* hybridization (FISH) indicated that *Sjc_0002003* was expressed throughout the body in both juvenile and mature worms (Fig 2A). Due to the lack of a single-cell RNA-seq atlas for *S. japonicum*, we examined the expression pattern of its homologous gene in *Schistosoma mansoni*, *Smp_171090*. The scRNA-seq data for *S. mansoni* showed that *Smp_171090* was expressed in nearly all cell types, with the highest levels observed in neoblasts, germline stem cells (GSCs), gut, parenchyma, flame cells, and neurons[12] (S1A Fig). We performed double FISH to further clarify the expression of *Sjc_0002003.* As shown, *Sjc_0002003* was co-localized with the somatic stem cell marker *nanos*2 and GSCs marker *nanos1*[12,13] (Fig 2B and 2C).

For *Sjc_0009272*, FISH revealed its expression throughout the body of both juvenile and adult *S. japonicum* (Fig 2D). Notably, its cell-type expression pattern differed from that of *Sjc_0002003*. In the single-cell atlas of *S. mansoni*, its homologous gene *Smp_340150* was expressed in most cell types, but with relatively low levels in neoblasts, GSCs, parenchyma, and flame cells[12] (S1B Fig). Additionally, double FISH with *nanos1* and *nanos2* revealed that *Sjc_0009272* was expressed in both GSCs and neoblasts (Fig 2E and 2F).

### *Sjc_0002003* and *Sjc_0009272* are required for cell proliferation in the parasite

Cell proliferation is essential for tissue maintenance in schistosomes[14,15]. Given that both *Sjc_0002003* and *Sjc_0009272* influence worm vitality and are expressed in somatic stem cells and GSCs (Figs 1C and 2), we investigated whether they modulate cell proliferation in the worms.

**Fig 3:**
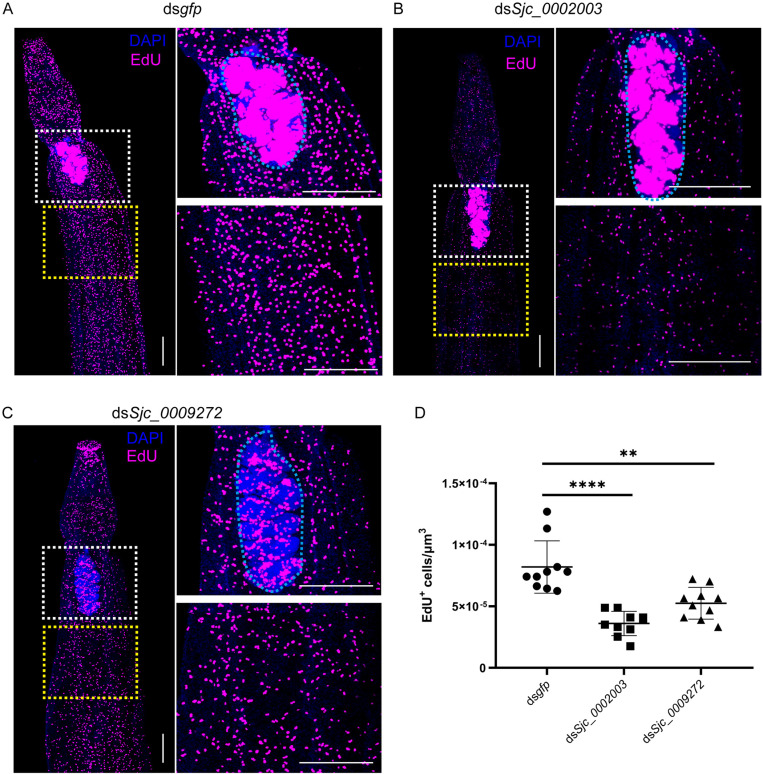
Silencing *Sjc_0002003* or *Sjc_0009272* result in reduced cell proliferation. (A-C) EdU labeling showing cell proliferation in control RNAi (A), *Sjc_0002003* RNAi (B) and *Sjc_0009272* RNAi (C) male parasites. Testes is in the white box. The blue dotted area is the testes. Yellow box indicates soma. Images are composed of multiple confocal stacks acquired from parasites 8 days after RNAi. Parasites were fixed after an overnight EdU labelling. Nuclei are shown in blue. EdU^+^ cells are in magenta. Scale bars: 200 μm. (D) Quantification of EdU^+^ cells per μm^3^ from the body in the yellow box. **n** = 10 worms. Each dot represents counts from a confocal stack taken from a single male parasite, with error bars representing 95% confidence intervals. Differences are statistically significant (****p < 0.0001, **p < 0.01, *t-test*).

Notably, both male and female worms can survive *in vitro* for several months[16,17]. However, while female worms retain their physical activity, their mature sexual organs may regress to an immature state under these conditions[18,19]. In this study, we aimed to investigate the roles of *Sjc_0002003* and *Sjc_0009272* in cell proliferation and germline cell development, focusing exclusively on male parasites. We used thymidine analog ethynyl deoxyuridine (EdU) to label the proliferative cells. Compared to controls, silencing *Sjc_0002003* significantly reduced cell proliferation in the bodies of adult males (Fig 3A, 3B, and 3D). Knockdown of *Sjc_0009272* also resulted in a significant decrease in the labeling of proliferating cells in the bodies of adult male worms (Fig 3A, 3C and 3D). Male testes contain a large number of dividing cells (e.g., germline stem cells) that could be labeled by EdU (Fig 3A). While the knockdown of *Sjc_0002003* strongly reduced proliferating cells in the body, proliferating cells of testes appeared unaffected. However, silencing *Sjc_0009272* dramatically decreased proliferating cells of testes (Fig 3A and 3C). These results demonstrate that *Sjc_0002003* and *Sjc_0009272* play important roles in regulating cell proliferation in *S. japonicum*.

### *Sjc_0002003* and *Sjc_0009272* are essential for the growth, development, and survival of *S. japonicum in vivo*

**Fig 4:**
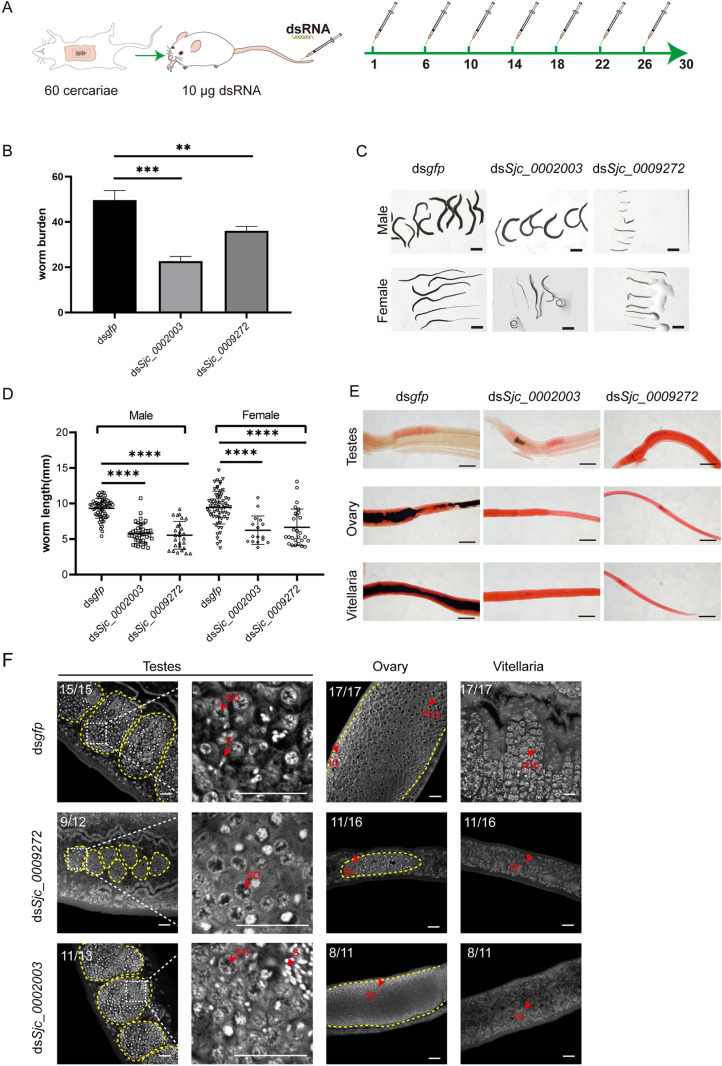
*Sjc_0002003* or *Sjc_0009272* RNAi affects the growth, development, and survival of *S. japonicum.* (A) Schematic diagram depicting the RNAi of schistosomula *in vivo*. On 1, 6, 10, 14, 18, 22, and 26 dpi, mice were injected in the tail vein with 10 μg of dsRNA targeting *Sjc_0002003* or *Sjc_0009272*. *gfp* dsRNA was used as a negative control. On day 30, worms were harvested from the mice. (B) Worm burden of the parasites recovered at 30 dpi in control (RNAi), *Sjc_0002003* (RNAi), and *Sjc_0009272* (RNAi) groups. (C) Morphological observation of worms after RNAi. Scale bars: 2000 μm. (D) Worm length of the parasites recovered at 30 dpi in control (RNAi), *Sjc_0002003* (RNAi), and *Sjc_0009272* (RNAi) groups. (E) Carmine staining showing the reproductive organs under light microscopy. Scale bars: 200 μm. (F) Confocol microscopy of the testes, ovaries, and vitellaria in control (RNAi), *Sjc_0002003* (RNAi), and *Sjc_0009272* (RNAi) worms. so, spermatocyte; s, sperm; io, immature oocyte; mo, mature oocyte; iv, immature vitelline cell, mv, mature vitelline cell. Scale bars: 20 μm. Error bars represent 95% confidence intervals, **n** ≥ 3. Differences are statistically significant (****p < 0.0001, ***p < 0.001, **p < 0.01, *t-test*).

Considering that both *Sjc_0002003* and *Sjc_0009272* were highly expressed in both sexes throughout the key developmental stages during the parasite maturation process from 14-28 dpi, we reasoned that they may play important roles in the growth or survival of the parasite in the host. To test it, we performed a 30-day *in vivo* RNAi on these two genes starting from the 1st day of infection, individually (Fig 4A). Compared to the control RNAi group, the worm burden in the *Sjc_0002003* RNAi and *Sjc_0009272* RNAi groups were significantly reduced with no sex bias (Figs 4B and S2A). Specifically, RNAi targeting *Sjc_0002003* reduced the worm burden by over 50%. Additionally, the growth of these worms was also inhibited in the *Sjc_0002003* and *Sjc_0009272* RNAi groups (Fig 4C). Both the male and female worms harvested from these two groups were significantly shorter than those in the control group (Fig 4C and 4D).

Carmine staining indicated that the testes in males, as well as the ovaries and vitellarium in females from the *Sjc_0002003* (RNAi) and *Sjc_0009272* (RNAi) groups, were less developed, especially in the worms treated with *Sjc_0009272* dsRNA (Fig 4E). We then examined the morphological changes in male and female reproductive organs under higher resolution using confocal laser scanning microscopy (CLSM). In the *Sjc_0002003* RNAi group, male testes still contained differentiated sperm, while the testes in the *Sjc_0009272* (RNAi) group exhibited severe defects (Fig 4F). The female sexual organs of both the *Sjc_0002003* (RNAi) and *Sjc_0009272* (RNAi) groups were undeveloped, lacking mature ovaries or vitellaria (Fig 4F). Notably, the *in vivo* RNAi of *Sjc_0009272* showed stronger inhibition of the reproductive organs in both male and female parasites compared to the *Sjc_0002003* RNAi group (Fig 4F). These results indicate that both *Sjc_0002003* and *Sjc_0009272* play critical roles in the growth, development, and survival of *S. japonicum*.

### *Sjc_0002003* is essential for the survival, maintenance of adult gonads, and oviposition in adult worms

**Fig 5:**
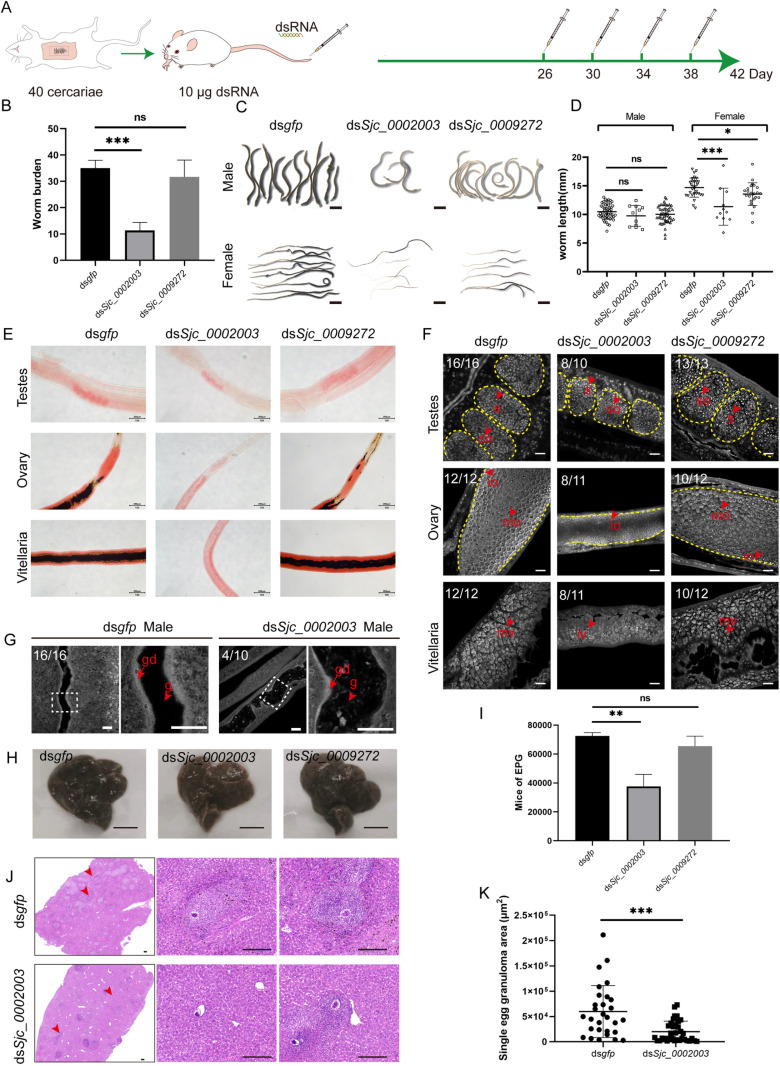
*In vivo* RNAi of *Sjc_0002003* and *Sjc_0009272* at the adult stage of *S. japonicum.* (A) Schematic diagram depicting RNAi of adult parasites *in vivo*. On 26, 30, 34, and 38 dpi, mice were injected in the tail vein with 10 μg of dsRNA targeting the specified genes. At 42 dpi, worms were harvested. (B) Worm burden of the parasites recovered at 42 dpi in control (RNAi), *Sjc_0002003* (RNAi), and *Sjc_0009272* (RNAi) groups. 3 mice per group. (C) Morphological observation of worms after RNAi. Scale bars: 2000 μm. (D) Worm length of the parasites by sex recovered at 42 dpi. (E) Gonad observation in female and male worms by Carmine staining. Scale bars: 200 μm. (F) Carmine staining of the testes, ovaries, and vitellaria in worms from different RNAi treatment. so, spermatocyte; s, sperm; io, immature oocyte; mo, mature oocyte; iv, immature vitelline cell; mv, mature vitelline cell. Scale bars: 20 μm. (G) Confocal imaging of the parasite gut in Carmine-stained males following *Sjc_0002003* silencing. g, gut; gd, gastrodermis. Scale bars: 25 μm. (H) Gross observations of the mouse liver from the ds*gfp* and ds*Sjc_0002003* treatment groups. Scale bars: 1 cm. (I) Egg count per gram of liver after RNAi. 3 livers per group. (J) Histological assessment of mouse liver by H&E (hematoxylin and eosin) staining. Scale bars: 200 μm. (K) Statistical analysis of the size of egg granuloma area after RNAi. 3 liver sections per group. Error bars represent 95% confidence intervals, **n** ≥ 3. ‘ns’ indicates no significant difference (*p* > 0.05). Differences are statistically significant (***p < 0.001, **p < 0.01, *p < 0.05, *t-test*).

To further evaluate the impacts of *Sjc_0002003* and *Sjc_0009272* on mature parasites, we performed *in vivo* RNAi on *S. japonicum* at 26 dpi (Fig 5A). Compared to the control, silencing *Sjc_0002003*—rather than *Sjc_0009272*—significantly reduced the worm burden with no sex bias (Figs 5B and S2B). Interestingly, the worm length of male parasites did not decrease in either dsRNA treatment group (Fig 5C and 5D). In contrast, the body length of females in both groups was significantly reduced (Fig 5C and 5D). Carmine staining revealed that the reproductive systems of both male and female parasites were degenerated in the *Sjc_0002003* (RNAi) group, without obvious changes in the *Sjc_0009272* (RNAi) group (Fig 5E). CLSM observation further confirmed this (Fig 5F). In addition, we found that some male worms in the *Sjc_0002003* (RNAi) group showed gut dilation (Fig 5G). Therefore, at the adult stage *in vivo*, the interference with *Sjc_0002003* strongly affects the survival of the parasite, and the remaining parasites exhibited severe reproductive defects, while the *Sjc_0009272* RNAi caused minor changes.

To further explore the effects of *Sjc_0002003* and *Sjc_0009272* on oviposition, we counted the deposited eggs in mouse livers. Compared to the control, the liver pathology in the *Sjc_0002003* (RNAi) group appeared weaker than the other groups, as indicated by the color of the liver (Fig 5H). This was further confirmed by evaluating of the number of liver eggs (Fig 5I). H&E staining showed that the size of the granuloma area formed by eggs in the livers of mice infected with *Sjc_0002003* (RNAi) worms was significantly smaller (Fig 5J and 5K).

These results suggest that *Sjc_0002003* is not only essential for the growth, development, and survival of juvenile worms, but also plays a crucial role in the survival and reproduction of adult worms.

### Potential regulatory mechanisms of *Sjc_0002003* in *S. japonicum*

**Fig 6:**
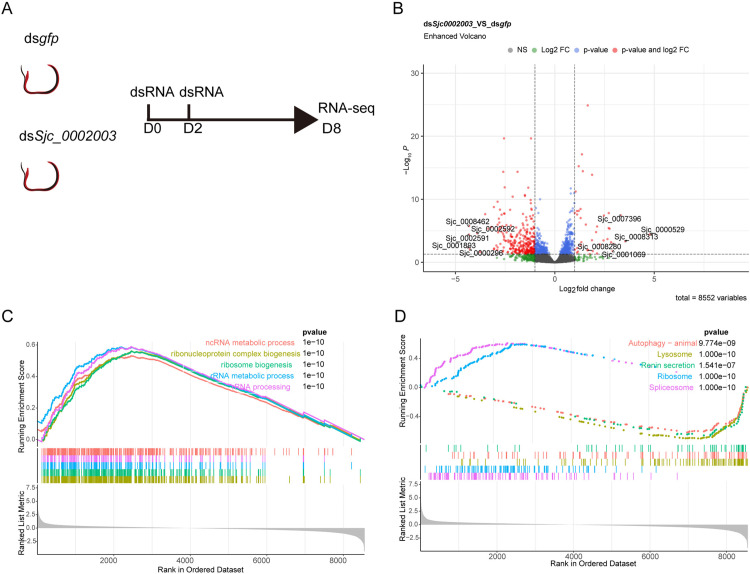
GO and KEGG pathway analysis results from GSEA. (A) RNAi strategy for RNA-seq: After 8 days of RNAi, paired worms were separated, and male parasites were harvested for RNA-seq. (B) Volcano plot illustrating differentially expressed genes (DEGs) in male parasites. DEGs were defined as |Log_2_ Fold Change| ≥1 and adjusted *P*-value< 0.05. (C) GSEA enrichment analysis of the expression data in males, focusing on Biological Processes. (D) GSEA enrichment analysis of the expression data in males, highlighting KEGG Pathway Analysis. Gene sets were considered significant only when |NES| > 1. The top 5 leading gene sets are displayed in the plot. GSEA, Gene Set Enrichment Analysis; GO, Gene Ontology; NES, normalized enrichment score.

Given the significant effects of *Sjc_0002003* on the parasite both *in vitro* and *in vivo* at different developmental stages, we further investigated the molecular network it may involve. Using RNA-seq, we profiled the differentially expressed genes at the time point when the phenotype emerged in male *S. japonicum* after 8 days of RNAi for *Sjc_0002003 in vitro* (Fig 6A). Compared to the control, there were 40 up-regulated (Log_2_ Fold Change ≥ 1, adjusted *P*-value < 0.05) and 288 down-regulated genes (Log_2_ Fold Change ≤ −1, adjusted *P*-value < 0.05) in *Sjc_0002003* (RNAi) males (Fig 6B and S7 Table).

To explore potential pathways and gene sets associated with *Sjc_0002003*, we conducted Gene Set Enrichment Analysis (GSEA) on the expression data (S8 Table). In the Gene Ontology (GO) analysis, 1,107 biological process (BP) terms were enriched (S9 Table). The top 5 enriched GO terms were associated with ribonucleoprotein complex biogenesis, ribosome biogenesis, rRNA metabolic process, rRNA processing, and RNA processing (Fig 6C and S9 Table). The top 5 KEGG pathways were enriched in spliceosome, ribosome, lysosome, autophagy – animal, and renin secretion (Fig 6D and S9 Table).

**Fig 7:**
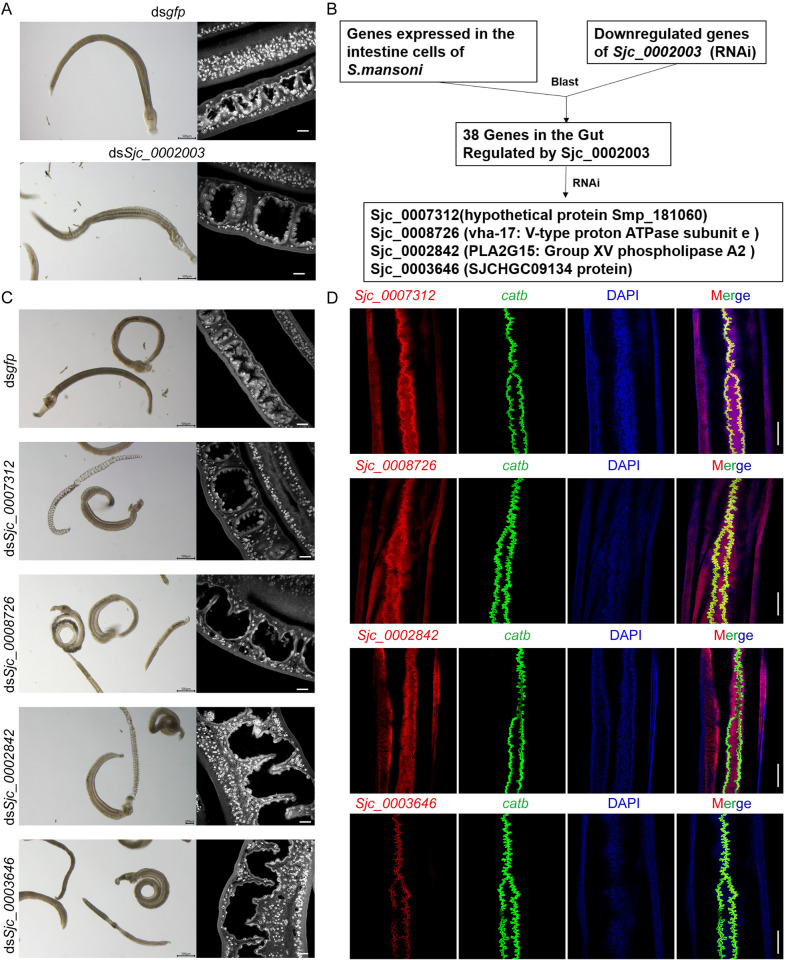
Sjc_0002003 modulates intestinal function through the regulation of intestinal genes. (A) Images of worms under light microscopy (left) and confocal images (right) of the Carmine-stained males after 12 days of RNAi treatment *in vitro*. Scale bars: 500 μm (left); 20 μm (right). (B) Schematic representation of the functional analysis of DEGs downregulated by *Sjc_0002003* RNAi. (C) Images of worms under light microscopy (left) and confocal images (right) of the Carmine-stained males after 30 days of RNAi treatment *in vitro*. Scale bars: 500 μm (left); 20 μm (right). (D) Colocalization of *Sjc_0007312*, *Sjc_0008726*, *Sjc_0002842* or *Sjc_0003646* with the intestinal marker *catb*. Scale bars: 200 μm.

Following *Sjc_0002003* knockdown, an apparent change in the worms was gut dilation (Fig 7A). Transmission electron microscopy (TEM) revealed that inhibition of *Sjc_0002003* expression led to severe disruption of the intestinal structure. Compared to controls, the microvilli in the intestines were sparse or absent, lipid droplets accumulated in the lumen, and localized intestinal cell lysis was evident (S3 Fig). To explore the molecular cause for this specific defect, we first identified the homologs of the 288 downregulated genes in the *Sjc_0002003* silenced parasites (Fig 6B and S7 Table) in *Schistosoma mansoni* and analyzed their average expression levels across different cell clusters (S10 Table) [12]. While these genes displayed a broad expression pattern, significant enrichment was observed in the gut cell clusters (S4 Fig and S10 Table)[12]. To explore the molecular cause for this specific defect, we firstly selected 81 intestinal genes that were significantly enriched in the gut cells from the *S. mansoni* single-cell atlas (http://www.collinslab.org/schistocyte/) and identified their homologous genes in *S. japonicum*. Surprisingly, 38 of these potential gut marker genes were significantly downregulated in the *Sjc_0002003* knockdown males (Fig 7B and S11 Table).

To identify the downstream genes that contribute to the gut dilation, we performed RNAi screening on these 38 candidate genes *in vitro* and found that RNAi of four genes—*Sjc_0007312* (hypothetical protein), *Sjc_0008726* (*vha-17*, encoding V-type proton ATPase subunit e), *Sjc_0002842* (*pla2g15*, encoding Group XV phospholipase A2), and *Sjc_0003646* (SJCHGC09134 protein)—could phenocopy the *Sjc_0002003* RNAi phenotype in the digestive tract (Figs 7C and S5A–S5D). As indicated by the carmine staining, silencing each of these four genes led to significant gut expansion compared to the control group (Fig 7C). Double FISH analysis using the intestinal marker *catb* (*cathepsin B-like cysteine proteinase*)[15] demonstrated that *Sjc_0007312*, *Sjc_0008726* and *Sjc_0003646* were highly expressed in the intestine of the parasite (Fig 7D). In the single-cell atlas of *Schistosoma mansoni*, the gene *Smp_166530* (homolog of *Sjc_0002842* in *Schistosoma japonicum*) is predominantly expressed in gut cells (S6A Fig) [12]. Although *Sjc_0002842* was not as highly expressed in the parasite intestine as the other three genes, it did co-localize with the intestine marker *catb* (Figs 7D and S6B). These results indicate that the downregulation of these genes, especially S*jc_0007312*, *Sjc_0008726*, *Sjc_0002842*, and *Sjc_0003646* may be key factors contributing to the intestinal phenotype observed after *Sjc_0002003* knockdown.

In Wendt *et al.*’s study, *Smhnf4* was shown to be required for the production of new gut cells, and loss of *Smhnf4* caused a dilated gut lumen[12]. We compared the RNA-seq data from *Sjc_0002003* knockdown (after 8 days) to the *Smhnf4* knockdown RNA-seq data and identified an overlap of 45 downregulated genes out of 121 significantly downregulated genes (S12 Table). Among these overlapping genes, 18 were enriched in intestinal cells, including the four genes (*Sjc_0007312*, *Sjc_0008726*, *Sjc_0002842*, and *Sjc_0003646*) identified in our study as associated with the gut dilation phenotype. Our RNA-seq data also showed that knocking down *Sjc_0002003* did not alter the expression of *Sjhnf4*[12]. These findings suggest that silencing *Sjc_0002003* may reduce the number of functional gut cells, leading to gut dilation. Thus, *Sjc_0002003* may play important roles in the gut homeostasis.

In summary, this study highlights the importance of UGs in the survival, development and reproduction of schistosomes, which calls for more attentions to these genes.

## Discussion

The genome of *S. japonicum* (SjV3) contains approximately 9,000 genes, with around 11% classified as “hypothetical proteins” or “proteins of unknown function”[10]. These proteins are identified mainly due to their expression from open reading frames, but lack proper annotation or functional validation[20]. In this study, we refer to them as UGs. Despite their prevalence in various organisms, research on these proteins is limited, yet they are believed to be crucial for the survival of pathogenic species. For example, protein LdBPK_070020 is hypothesized to be vital for *Leishmania donovani*[21], and the hypothetical protein b427.10.13790 has been implicated in cytokinesis in *Trypanosoma brucei*[22]. We hypothesize that UGs also play significant roles in schistosomes. To explore this, we prioritized 126 UGs that are highly expressed during the sexual developmental stages of *S. japonicum*, identifying 8 that are essential for the parasite’s vitality. However, the importance of the remaining UGs cannot be overlooked, as they may function in other developmental stages of the parasite.

The *in vitro* RNAi methodology serves as a rapid and effective tool for screening functional genes in schistosomes. This approach allowed us to quickly narrow down the target UGs from hundreds of genes by observing physiological changes in the parasites under light microscopy. However, we must acknowledge the potential for false negatives among the genes that did not display a phenotype, as RNAi efficiency can vary, leading to incomplete knockdown for some genes. The phenotypes we defined in this study were visually apparent changes in the worms, yet other effects, such as stem cell loss, might not produce obvious changes *in vitro*, as noted by Wang *et al*.[4]. Additionally, *in vitro* conditions lack stressors from the host, like immune responses and blood flow, suggesting that genes showing minimal defects in *in vitro* RNAi may exhibit significant phenotypes *in vivo.*

Comparative analysis with human and mouse genome data shows that most proteins encoded by UGs in *S. japonicum* lack homologs in their mammalian hosts (S2 and S3 Tables). Notably, Sjc_0002003 and Sjc_0009272, which displayed significant phenotypes in *in vitro* RNAi assays, are conserved across trematodes. SMART analysis indicated that protein Sjc_0002003 (GenBank: KAH8855387.1) lacks any conserved domains (S7A Fig), while Sjc_0009272 (GenBank: KAH8856685.1) possesses a signal peptide and transmembrane region (S8A Fig). Amino acid sequence alignment and phylogenetic analysis revealed that both proteins share highly conserved sequences with other *Schistosoma* species (S7A and S8B Figs). Although highly similar homologous proteins were found in other trematodes, no homologs were detected in *Homo sapiens* or *Mus musculus* (S7B, and S8C Figs), underscoring their conserved roles in trematodes.

Although Sjc_0002003 and Sjc_0009272 are annotated as UGs due to the lack of homologs in their mammalian hosts based on our BLAST analysis, we noticed that their homologous genes in *S. mansoni*, Smp_171090 (a homolog of Sjc_0002003), is annotated as an Anaphase-promoting complex subunit CDC26, while Smp_340150 (a homolog of Sjc_0009272) is annotated as an ABC transporter subunit. Upon detailed analysis, we performed a BLASTp search on NCBI using the encoded product of Sjc_0002003 against model organisms, including humans, which returned “No significant similarity found.” We then conducted a one-to-one comparison between the Sjc_0002003 protein and Human CDC26 (*Hs*CDC26) and observed some conserved residues, although the overall identity was low at 24% (S9A Fig). Using NCBI’s CD-search tool (https://www.ncbi.nlm.nih.gov/Structure/cdd/wrpsb.cgi), we identified a conserved domain in *Hs*CDC26 as the “CDC26 family anaphase-promoting complex subunit” (S9B Fig). However, no conserved domains were detected in Sjc_0002003 or its homologous protein, Smp_171090, in *S. mansoni*. Based on these findings, annotating Sjc_0002003 or Smp_171090 as an anaphase-promoting complex subunit CDC26 lacks sufficient supporting evidence. A similar analysis of Sjc_0009272 revealed no significant similarity to model organisms via BLASTp, and no conserved domains were identified using the CD-search tool. These results suggest that the annotations for schistosomes in public databases may be derived from automated, machine-assisted annotations that require manual review.

Our *in vitro* RNAi experiments showed that silencing *Sjc_0002003* and *Sjc_0009272* significantly reduced the activity of adult worms. However, *in vivo* RNAi targeting these genes during the adult stage resulted in distinct phenotypes: knockdown of *Sjc_0002003* significantly decreased worm burden, while *Sjc_0009272* RNAi had no impact on worm burden and caused only minor changes in reproductive organs. This clear difference in RNAi phenotypes is primarily attributed to varying RNAi efficiencies under the two conditions. For *Sjc_0002003*, RNAi efficiency *in vivo* matched that observed *in vitro* (S10A Fig). In contrast, the RNAi efficiency for *Sjc_0009272 in vivo* was significantly lower than *in vitro* (S10B Fig). This suggests that complex host conditions can affect RNAi efficiency, which appears to be dsRNA-dependent. Therefore, careful evaluation of RNAi efficiency is crucial in these contexts. Additionally, *in vivo* RNAi experiments initiated in the early stages after infection demonstrated that worm growth was inhibited in the *Sjc_0009272* RNAi groups (Fig 4C). This phenotype could be attributed to either slowed parasite growth or complete growth arrest. Given that worm burden was also reduced following *Sjc_0009272* knockdown, we hypothesize that the surviving short worms ceased growing, possibly due to lower RNAi efficiency, which led to developmental defects preventing them from reaching the adult stage rather than eliminating them entirely.

The detachment phenotype observed *in vitro* adults would possibly result in the clearance of these parasites *in vivo*, as the worms would lose their ability to attach to blood vessels, which is essential for migration. This loss, combined with other defects caused by RNAi that may reduce the worms’ tolerance to host stress, would likely cause them to be flushed into the liver, as suggested by Collins *et al.*[23]. Consistent with this, we observed dead worms in the host liver by examining mouse liver sections stained with H&E in the *Sjc_0002003* (RNAi) group (S11 Fig). These findings strongly suggest that RNAi treatment targeting *Sjc_0002003* disrupts critical processes necessary for worm survival *in vivo*, leading to their inability to thrive in the host environment and their eventual clearance via the liver. Further investigation is required to determine the precise mechanisms by which *Sjc_0002003* knockdown induces detachment and compromises worm survival, which could provide valuable insights into novel therapeutic strategies targeting schistosome survival and attachment mechanisms.

Among the 8 UGs identified in our screening, *Sjc_0002003* RNAi resulted in the most pronounced phenotype, confirmed through both *in vitro* and *in vivo* assays. We focused on the gut dilation phenotype and identified 4 genes related to this specific intestinal defect from the downregulated genes enriched or expressed in the gut. We speculated that knocking down *Sjc_0002003* or the four genes may disrupt the normal function of gut cells, potentially leading to the loss of functional cells and, subsequently, gut dilation. Two of these genes, an ATPase subunit and a phospholipase A2, share a common feature: both are involved in lysosome function. Specifically, the homolog of the V-type proton ATPase subunit e (Sjc_0008726) is a protein component of the larger V-ATPase complex located on lysosomal membranes in other organisms, where it plays a crucial role in maintaining lysosomal acidity by actively pumping protons (H⁺) into the lumen using energy from ATP hydrolysis[24]. Similarly, the homolog of Group XV phospholipase A2 (Sjc_0002842) is known as lysosomal phospholipase A2 (LPLA2), encoded by the PLA2G15 gene. It is primarily located within lysosomes in other organisms[25]. Considering that lysosomes function as the digestive system of cells and that the schistosome gut plays a vital role in reusing host nutrients, we hypothesize that the loss of function of these two genes may impair lysosomal function, further disrupting gut functionality. For the other genes, due to their low similarity to well-studied homologs in other organisms, we currently lack insights into their roles. Nevertheless, further studies are necessary to address these questions. As noted by Wendt *et al.*[12], inhibiting the expression of gut genes had a minimal impact on adult worm viability *in vitro*. Additionally, since *Sjc_0002003* is expressed across all cell types in the parasite, we hypothesized that the strong physiological disruption caused by *Sjc_0002003* RNAi is due to a comprehensive defect. Since *Sjc_0002003* is an unknown gene with no prior clues regarding its function, identifying enriched GO terms and KEGG pathways provides a general overview of the changes resulting from its silencing. As shown in S9 Table, there are over 1,000 enriched GO terms and multiple KEGG pathways, indicating that knocking down *Sjc_0002003* has a broad impact on the parasite. Our RNA-seq analysis revealed the downregulation of autophagy-related genes (Fig 7D), which are crucial for maintaining cellular homeostasis and stress resistance[26]. Previous studies have indicated that autophagy inhibitors adversely affect worm fitness, egg production, and the morphology of gonads and intestines in *S. mansoni*[27]. Therefore, the downregulation of autophagy-related genes may further contribute to the reduced viability of worms treated with *Sjc_0002003* RNAi.

In summary, we identified 8 non-host homologous proteins that influence the physiological activities of *S. japonicum in vitro*, focusing particularly on the biological functions of the UGs *Sjc_0002003* and *Sjc_0009272*. Our results underscore the significance of these two genes for the development and survival of *S. japonicum*. Importantly, these findings indicate that UGs are functional in schistosomes, suggesting that further exploration of these genes is warranted.

## Methods

### Ethics statement

Animals manipulation was conducted in strict compliance with the animal care and use guidelines established by Fudan University. All procedures were approved by the Animal Care and Use Committee of Fudan University (Fudan IACUC 201802158S) to ensure the ethical and responsible treatment of the animals.

### Parasites and animals

Cercariae of *S. japonicum* (Anhui isolate) required for this study were released from infected snails (*Oncomelania hupensis*), provided by the Institute for Parasitic Disease Prevention and Control (CDC) of the Chinese Center for Disease Control and Prevention. Female BALB/c mice, aged 6 weeks and weighing 16-20 g, were purchased from Shanghai Jiesijie Laboratory Animal Co., Ltd. (Shanghai, China). For the *in vitro* experiments, mice were infected with approximately 150 cercariae, while for the *in vivo* experiments, they were infected with 60 ± 2 or 40 ± 2 cercariae. Mice were euthanized by carbon dioxide asphyxiation at 30 or 42 dpi. Worms were collected from infected mice by perfusion using a saline solution containing sodium heparin[28].

### RNA extraction, cDNA synthesis and qPCR analysis

RNA was extracted using AG RNAex Pro RNA Reagent (AG21101, Accurate Biotechnology, Changsha, Hunan, China) according to the manufacturer’s guidelines, and then reverse transcribed to cDNAs using the Evo M-MLV RT Premix Kit (AG11711, Accurate Biotechnology, Changsha, Hunan, China).

All qPCR reactions were performed on a LightCycler 96 (Roche, Basel, Switzerland) using 2× SYBR Green Pro Taq HS Premix (AG11701, Accurate Biotechnology, Changsha, Hunan, China) and 0.5 μL of each primer. PCR amplification was performed with an initial denaturation at 95 °C for 5 minutes, followed by 40 cycles of 95 °C for 5 seconds and 60 °C for 30 seconds. A melt curve analysis from 60 °C to 95 °C was used to verify product specificity. Using *Sjpsmd4* (26S proteasome non-ATPase regulatory subunit 4, GenBank ID: FN320595) as the internal reference, the 2^−ΔCt^ method was employed for relative quantification of mRNA expression levels. The qPCR primer sequences used in this study are shown in S13 Table.

### dsRNA synthesis

For the design of double-stranded RNA (dsRNA) primers, the *S. japonicum* genome (SjV3) was referenced to obtain the coding sequence (CDS) of the target genes, using Primer Premier 6.0 for primer design. The target sequence primer was designed at the 5’ end of the gene, with a length of approximately 450~600 bp. For coding sequences less than 500 bp, primers were designed to cover as much of the transcript as possible. The primers used to synthesize dsRNA included a T7 sequence (TAATACGACTCACTATAGGGAGA) at the 5’ end of each oligo. The dsRNA primer sequences used in this study are shown in S14 Table. dsRNA was synthesized based on an established method previously described[29]. DNA templates were amplified from cDNA of male and female adult worms using a 2×Hieff PCR Master MIX (with Dye) kit (YEASON, Shanghai, China) and confirmed by Sanger Sequencing. Following the manufacturer’s instructions, dsRNA was synthesized using the MEGAscript T7 High Yield Transcription Kit (Invitrogen, USA). Finally, the dsRNA was aliquot and store at -20°C for later use.

### RNAi *in vitro*

A total of 3 mL of DMEM medium (10% FBS, 1% penicillin - streptomycin -amphotericin B solution) was added to each well of a 12-well cell culture plate. Five pairs of well-conditioned mature *S. japonicum* were placed in each well. Referring to the interference procedure of Wang *et al.*[4], dsRNA (30 μg/mL) was added. The culture medium was refreshed every 2 days. During the RNAi screening, we monitored the parasites every other day for 30 days, recording the first day on which abnormalities were observed compared to the control group, as well as the phenotypes on the final day.

### RNAi *in vivo*

Infected mice were injected intravenously via the tail with 10 μg of dsRNA per injection[28]. Control mice were injected with dsRNA of *gfp,* a green fluorescent protein from *Aequorea Victoria* that is not present in *S. japonicum*[30].

### Phylogenetic analysis and multiple sequence alignment

The keywords “hypothetical protein” and “protein of unknown function” were used to identify genes encoding uncharacterized proteins in the *S. japonicum* genome. The amino acid sequences of these proteins were compared with protein datasets from the *Homo sapiens* and *Mus musculus* genomes using BLAST (https://www.ncbi.nlm.nih.gov).

Protein sequences were compared using the NCBI database (https://www.ncbi.nlm.nih.gov) to identify homologous sequences of Sjc_0002003 in other species. The selected comparison species included *Schistosoma haematobium*, *Schistosoma mansoni*, *Schistosoma intercalatum*, *Schistosoma spindale*, *Schistosoma bovis*, and *Schistosoma turkestanicum*, as well as other fluke species (such as *Fasciola hepatica*, *Fasciola gigantica*, *Fasciolopsis buski*, and *Echinostoma caproni*), tapeworms (such as *Echinococcus granulosus*) and schistosomiasis-related hosts (such as *Homo sapiens*). The retrieved homologous sequences were aligned using ClustalW in MEGA 7, and phylogenetic analysis was performed using the Neighbor-Joining (NJ) method. The evolutionary tree was then refined using iTOL (https://itol.embl.de/).

### RNA-seq

Paired males were collected and washed three times with 1×PBS (pH7.4). After quick freezing in liquid nitrogen, the samples were sent to Novagene Technology Corporation (Beijing, China) for RNA extraction, library construction and sequencing. RNA was extracted using the phenol-chloroform method, and the RNA quality was assessed using a Nanodrop ND-2000 and Agilent 2100. According to the instructions, the NEBNext Ultra RNA Library Prep Kit was used to construct a sequencing library and sequencing was performed on the Illumina Nova 6000 platform. The FASTQC program (https://www.bioinformatics.babraham.ac.uk/projects/fastqc/) performed quality control on Raw data. FASTP v.0.20.1 was used to remove low-quality reads and adapters (the parameters are -q 15 -u 40 -n 5 -l 15) to obtain clean reads[31]. Clean reads were mapped to the *S. japonicum* genome (SjV3) using HISAT2 v2.1.0[32]. Transcript abundance was assessed using FeatureCounts[33], and DEseq2 was employed to analyze gene expression differences[34]. GSEA enrichment was performed using the R package cluster Profiler[35,36]. Based on the single-cell atlas of *Schistosoma mansoni*[12], the distribution of homologs of the down-regulated genes across various cellular clusters was analyzed. The *AverageExpression()* function was used to calculate the mean expression levels of these homologous genes in different cell clusters. The expression values were then normalized using the formula Log_10_(AverageExpression), and the results were visualized with GraphPad Prism 8.0.

### Fluorescence *in situ* hybridization

Fluorescence *in situ* hybridization (FISH) was performed according to previously described methods[37]. A T7 promoter was added to the 5’ end of the downstream primer. The probe primer sequences are shown in S15 Table. PCR amplification was used to obtain the target fragment DNA. Using the target DNA fragment as a template, probes were synthesized using DIG RNA Labeling Mix or Fluorescein RNA Labeling Mix (Roche, Germany). Double FISH experiments were conducted following the methods described by Wang *et al.*[18].

### EdU labeling and detection

Ten μM EdU (Invitrogen, USA) was added to the worm culture medium *in vitro* and incubated in a 37°C incubator for 4 hours. The method of Wang *et al.*[38] was followed to process the worms after EdU pulse and detect EdU incorporation. Fluorescently labeled samples were imaged using a Nikon A1 Laser Scanning Confocal Microscope (Nikon, Japan). All images of fluorescently labeled samples represent maximum intensity projections. To count EdU^+^ cells, cells were manually counted in the maximum intensity projection derived from the confocal stack. To normalize between samples, cell counts were divided by the total volume of the stack. All plots and statistical analyzes were performed using GraphPad Prism.

### Morphological observation of schistosomes

Fresh worms were fixed in the AFA solution (24% formaldehyde, 50% ethanol, 4% acetic acid) for morphometric and morphological analyses with carmine staining, as previously described[39]. The worms were mounted on the glass slides using Canadian resin. After the resin solidified completely, bright-field analysis was performed with an ordinary upright microscope, followed by observations of changes in the gonadal organs of the worm using a Nikon A1 Laser Scanning Confocal Microscope (Nikon, Japan).

### H&E staining

The liver of each mouse was fixed with paraformaldehyde and stained with hematoxylin and eosin (H&E) to assess granulomas formation, as previously described[40]. Observations and photographs were taken under an ordinary upright microscope.

### Liver egg counting

The mouse liver was weighed (Lg) and 35 ml of 5% sodium hydroxide was added. The mixture was digested on a shaker at 37 °C and 220 rpm to obtain a homogeneous solution. Ten μL of the above solution was observed and counted under an ordinary upright microscope. Each sample was counted 10 times, and the average (E) was calculated. Each group had at least three biological replicates. The number of liver eggs (EPG) was calculated using the formula: EPG = E × 100 × 35/ Lg.

### Statistical analysis

GraphPad Prism 8.0 (GraphPad Software, USA) was used for all statistical analyses. Comparisons between groups were performed using an unpaired two-tailed parametric *t* test. *P* values < 0.05 were considered significant.

## Supporting information

S1 Fig UMAP projections showing the expression of *Sjc_0002003* and *Sjc_0009272* homologs in *S. mansoni* cell types.
(A-B) UMAP projections depicting the expression profiles of Smp_171090 (homologous to Sjc_0002003 in *S. japonicum*) and Smp_340150 (homologous to Sjc_0009272 in S. japonicum) in different cell clusters in virgin female *S. mansoni* (top), adult female (middle), and adult male (bottom).
(TIF)

S2 Fig Worm burden of the males and females after *in vivo* RNAi.
(A-B) Worm burden of the males and females recovered at 30 dpi (A) and 42dpi (B) in control (RNAi), Sjc_0002003 (RNAi), and Sjc_0009272 (RNAi) groups. Three mice per group. Error bars represent 95% confidence intervals. Differences are statistically significant (***p < 0.001, **p < 0.01, *p < 0.05, *t-test*).
(TIF)

S3 Fig Observation of the intestinal structure in male parasites following *Sjc_0002003* inhibition under Transmission electron microscopy.
Male parasites were treated with either control or Sjc_0002003 dsRNA for 8 days before TEM. The intestine region is indicated by yellow dashed line. N, nucleus; Nu, nucleolus; mv, microvillus; L, lumen; LD, lipid droplet.
(TIF)

S4 Fig Cellular cluster distribution of the *Schistosoma mansoni* homologs of the down-regulated genes in *Sjc_0002003* (RNAi).
(TIF)

S5 Fig qPCR analysis detecting RNAi efficiency for four genes causing intestinal phenotypic changes.
(A-D) Relative expression levels of *Sjc_0007312* (A), *Sjc_000872*6 (B), *Sjc_0002842* (C) and *Sjc_0003646* (D) after dsRNA treatment for 10 days in males. *Sjpsmd4* was used as the internal reference. Three biological replicates were performed. Error bars represent 95% confidence intervals. Differences are statistically significant (****p < 0.0001, ***p < 0.001, **p < 0.01, *t-test*).
(TIF)

S6 Fig Sjc_0002842 is expressed in gut cells.
(A) UMAP projections depicting the expression profiles of Smp_166530 (homologous to Sjc_0002842 in *S. japonicum*) in different cell clusters in adult male (left) and female (right) *S. mansoni*. (B) Double FISH of *Sjc_0002842* with the intestinal marker *catb*. Scale bars: 25 μm.
(TIF)

S7 Fig Sjc_0002003 is evolutionally conserved within the *Trematoda.
* (A) Sequence alignment of Sjc_0002003 from *S. japonicum* with homologs from other species. The sequence ID includes the Genbank no. The Genbank no. displayed in white characters on a black background is Sjc_0002003. (B) Phylogenetic analysis of Sjc_0002003 and its homologs. The sequence ID includes the Genbank no.+ and species name. Sjc_0002003 is labelled in purple.
(TIF)

S8 Fig Sjc_0009272 is evolutionarily conserved within the *Trematoda.
* (A) Protein domains of Sjc_0009272. (B) Sequence alignment of Sjc_0009272 from *S. japonicum* with homologs from other species. The sequence ID includes the Genbank no. The Genbank no. displayled in white characters on a black background is Sjc_0009272. (C) Phylogenetic analysis of Sjc_0009272 and its homologs. The sequence ID includes the Genbank no. and species name. Sjc_0009272 is labelled in purple.
(TIF)

S9 Fig Sjc_0002003 and Sjc_0009272 have no homology identified in *Homo sapiens*. ( A) Needleman-Wunsch alignment of Sjc_0002003 and *Hs*CDC26. (B) Identification of conserved domains on *Hs*CDC26.
(TIF)

S10 Fig Efficiency of RNA interference for *Sjc_0002003* and *Sjc_0009272.
* (A-B) Following dsRNA treatment and RNA-seq analysis, males showed a Log2 fold change in Sjc_0002003 (A) and Sjc_0009272 (B). *In vitro*, 8D worms were obtained after 8 days of dsRNA treatment. At 30 dpi, 30D worms were harvested *in vivo* from mice that had been injected via the tail vein with 10 μg of dsRNA targeting the specified genes on 1, 6, 10, 14, 18, 22, and 26 dpi. At 42 dpi, 42D worms were harvested *in vivo* from mice injected via the tail vein with 10 μg of dsRNA targeting the specified genes on 26, 30, 34, and 38 dpi.
(TIF)

S11 Fig Worms in the liver of mice treated with Sjc_0002003 (RNAi). In the Sjc_0002003(RNAi) group, the worms deposited in the liver was surrounded by neutrophils and lymphocytes. Scale bars: 50 μm.
(TIF)

S1 Table Uncharacterized genes in *S. japonicum.
* (XLSX)

S2 Table List of uncharacterized genes in *S. japonicum* that show similarity to *Homo sapiens* proteins.
(XLSX)

S3 Table List of uncharacterized genes in *S. japonicum* that show similarity to *Mus musculus* proteins.
(XLSX)

S4 Table TMM normalization counts of UGs and top 100 uncharacterized genes with high transcript levels at each stage by sex from 14-28 dpi.
(XLSX)

S5 Table Information on 126 genes selected for RNAi screening.
(XLSX)

S6 Table Details of 8 uncharacterized genes showing detachment and morphological changes.
(XLSX)

S7 Table Differentially expressed genes in male parasites following *Sjc_0002003* RNAi treatment.
(XLSX)

S8 Table Expression data of male parasites following *Sjc_0002003* RNAi treatment.
(XLSX)

S9 Table GSEA enrichment analysis of expression data after *Sjc_0002003* RNAi.
(XLSX)

S10 Table Average expression levels of homologs of downregulated genes in *Sjc_0002003* (RNAi) across various cell clusters of *S. mansoni.
* (XLSX)

S11 Table Thirty-eight differentially expressed genes homologous to intestinal genes of *S. mansoni.
* (XLSX)

S12 Table Overlap of down-regulated genes in male parasites from the *Sjc_0002003* (RNAi) and *Smhnf4* (RNAi) groups.
(XLSX)

S13 Table qPCR primer sequences. (XLSX).

S14 Table dsRNA primer sequences.
(XLSX)

S15 Table Probe primer sequences.
(XLSX)
