# Supplementary figures and images for: RNAi screening of uncharacterized genes identifies promising druggable targets in Schistosoma japonicum
Source: PLoS Pathog. 2025 Mar 28;21(3):e1013014. doi: 10.1371/journal.ppat.1013014 (PMC11977999; doi:10.1371/journal.ppat.1013014)

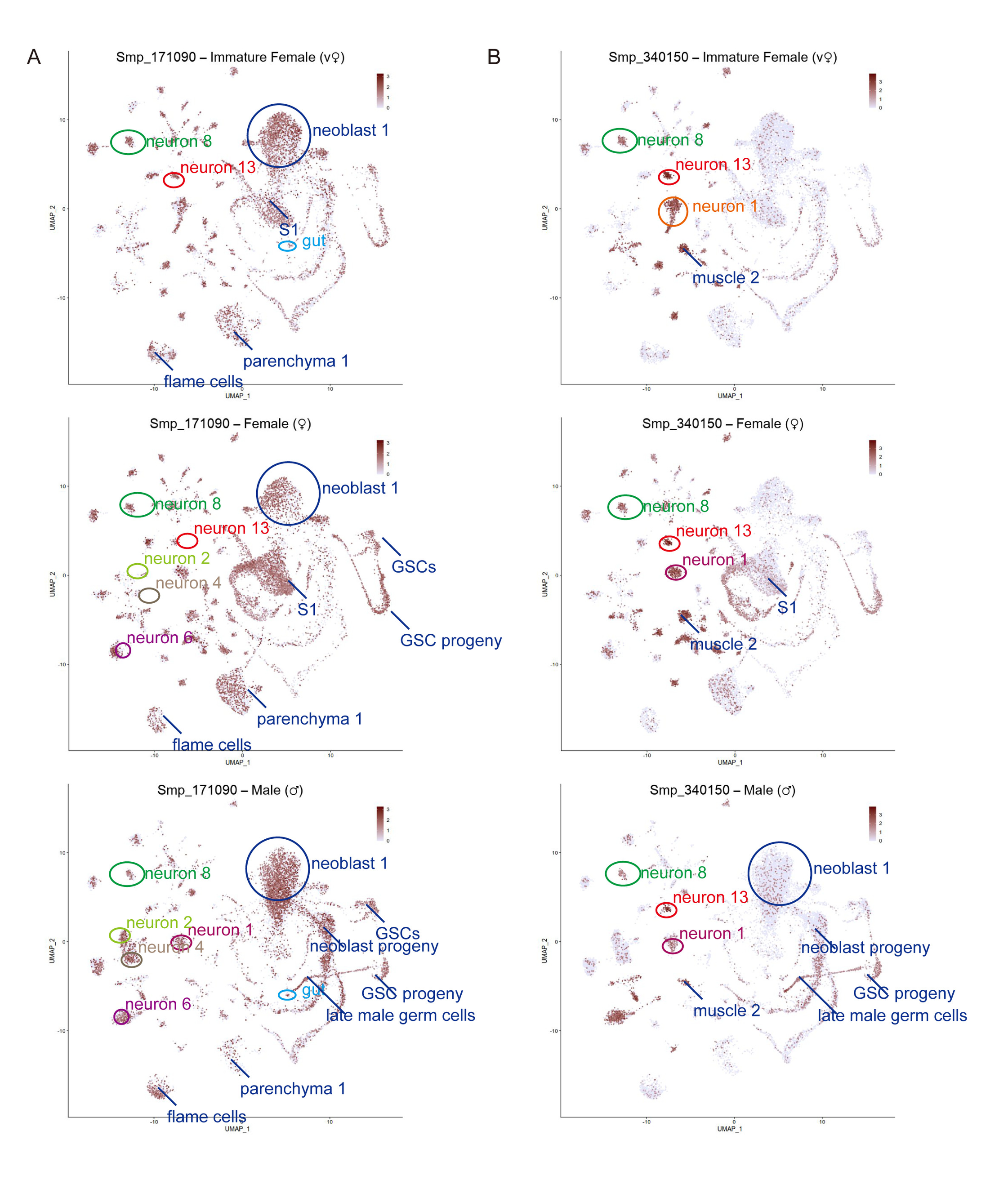

Supplement: S1 Fig — (A-B) UMAP projections depicting the expression profiles of Smp_171090 (homologous to Sjc_0002003 in S. japonicum) and Smp_340150 (homologous to Sjc_0009272 in S. japonicum) in different cell clusters in virgin female S. mansoni (top), adult female (middle), and adult male (bottom). (TIF) [file ppat.1013014.s001.tif]

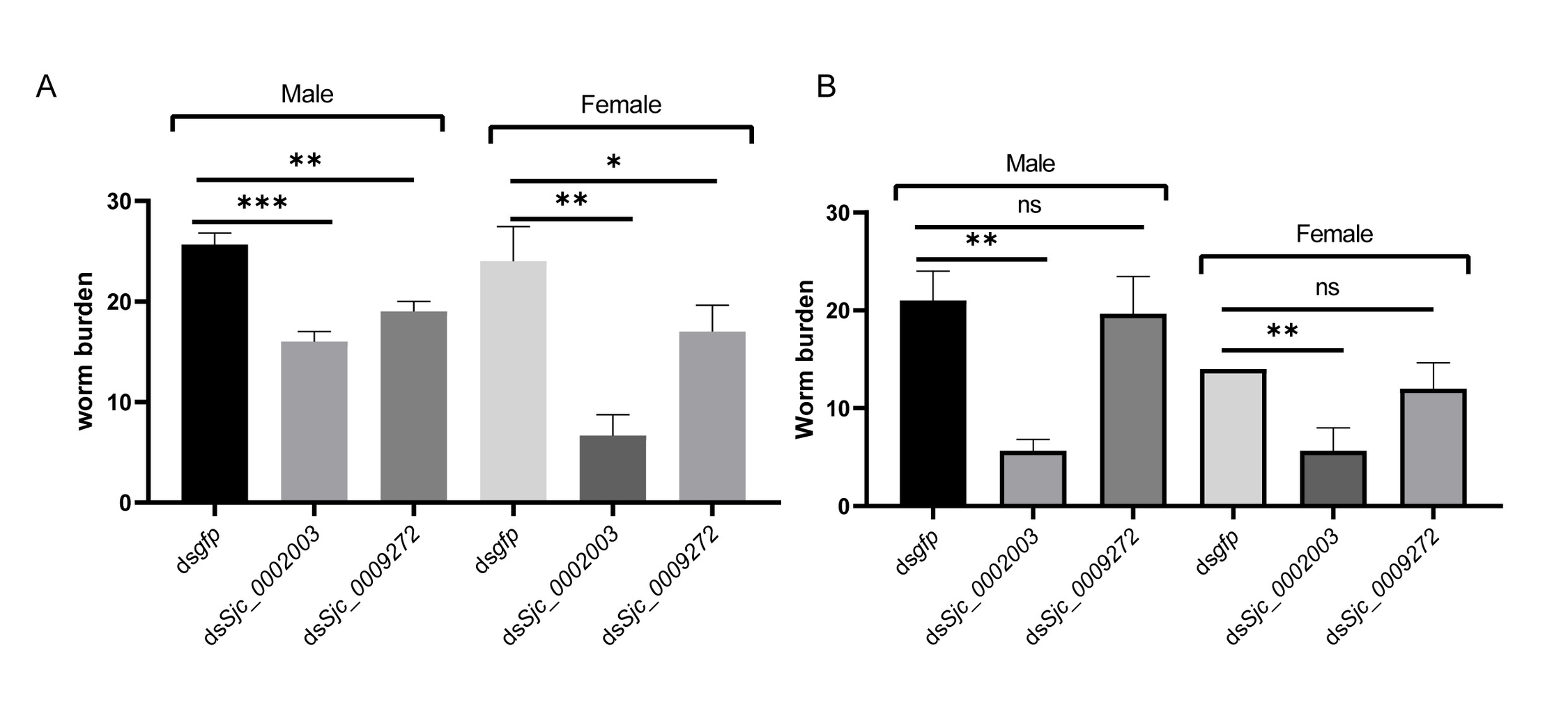

Supplement: S2 Fig — (A-B) Worm burden of the males and females recovered at 30 dpi (A) and 42dpi (B) in control (RNAi), Sjc_0002003 (RNAi), and Sjc_0009272 (RNAi) groups. Three mice per group. Error bars represent 95% confidence intervals. Differences are statistically significant (***p < 0.001, **p < 0.01, *p < 0.05, t-test). (TIF) [file ppat.1013014.s002.tif]

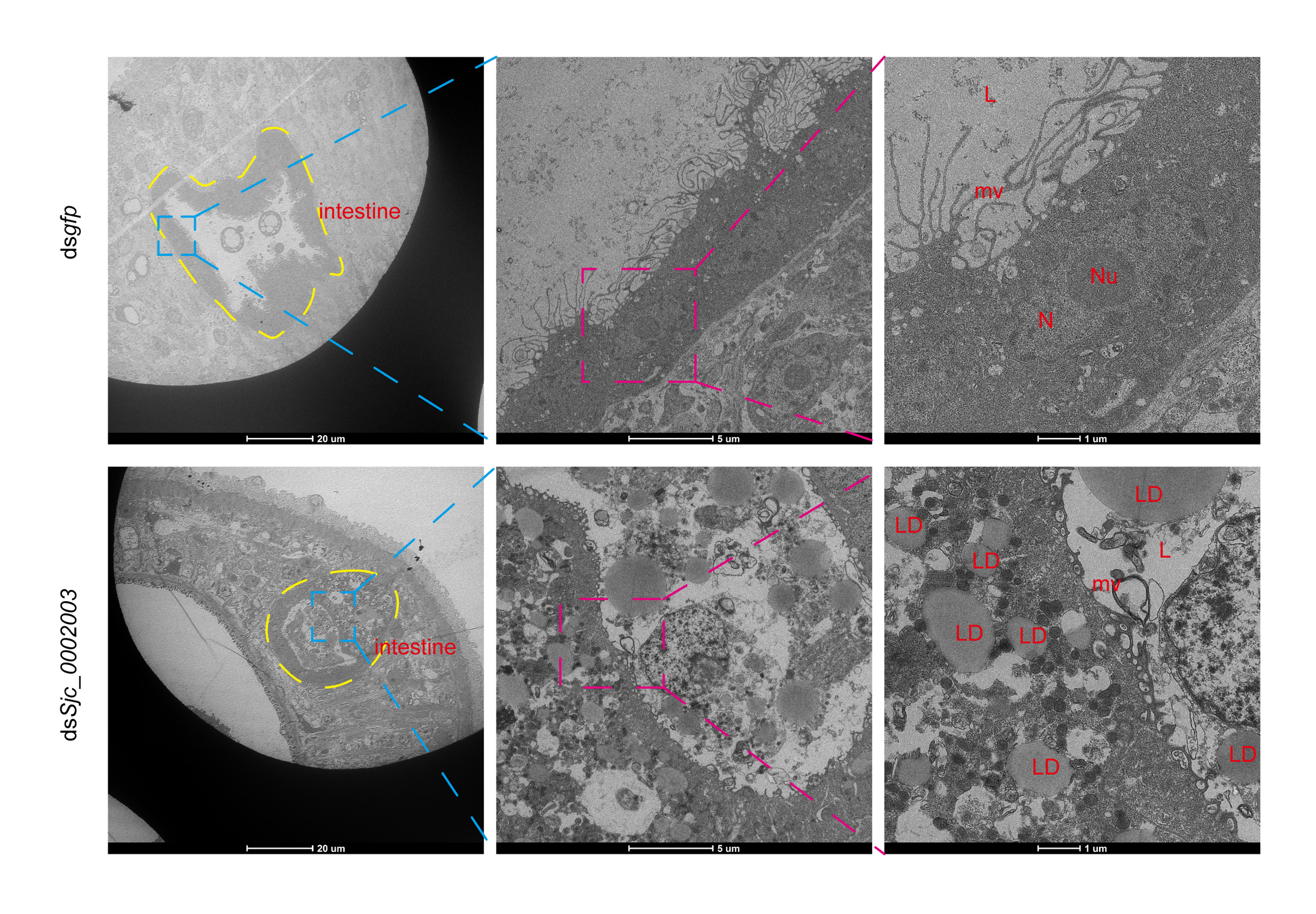

Supplement: S3 Fig — Male parasites were treated with either control or Sjc_0002003 dsRNA for 8 days before TEM. The intestine region is indicated by yellow dashed line. N, nucleus; Nu, nucleolus; mv, microvillus; L, lumen; LD, lipid droplet. (TIF) [file ppat.1013014.s003.tif]

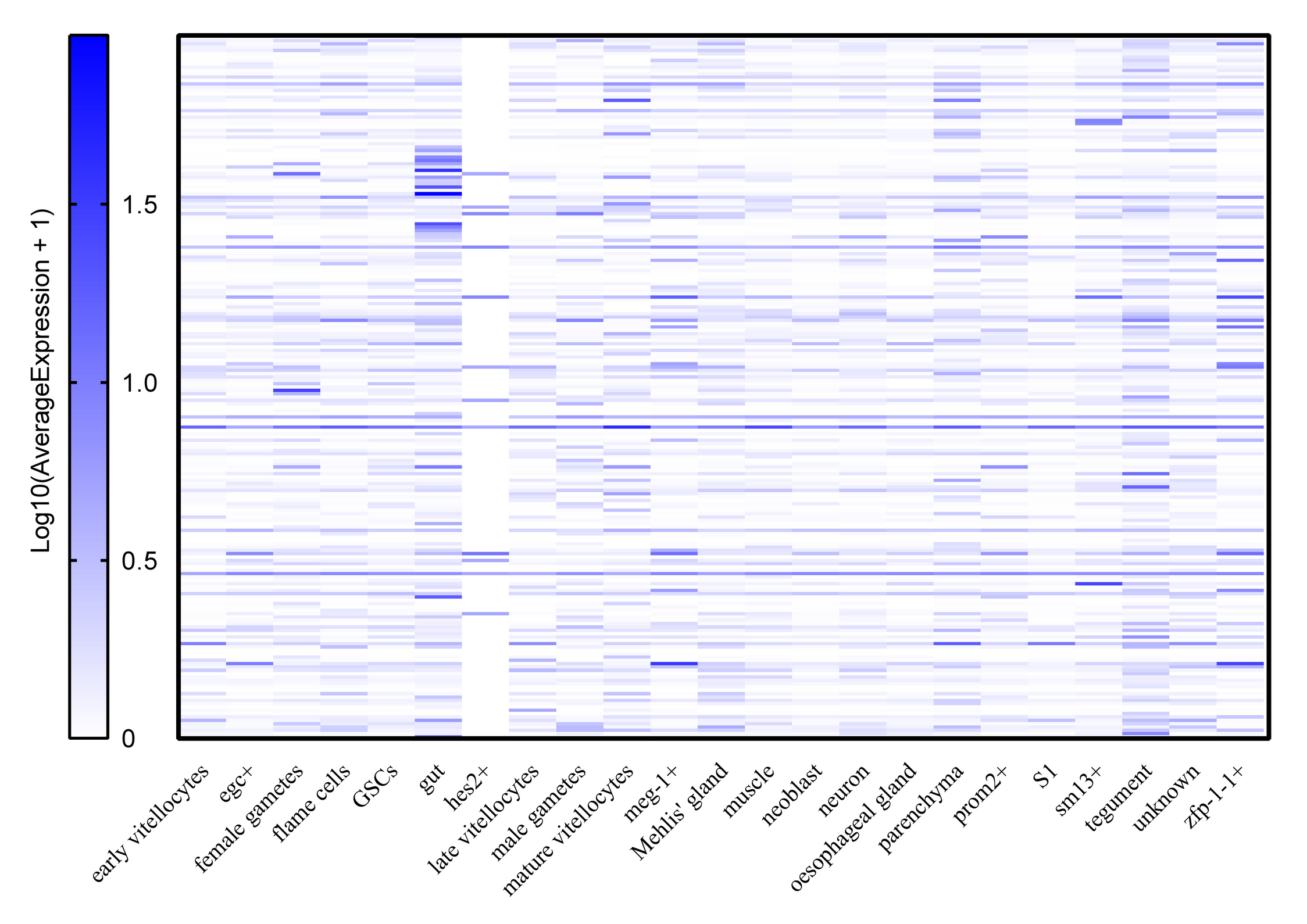

Supplement: S4 Fig — (TIF) [file ppat.1013014.s004.tif]

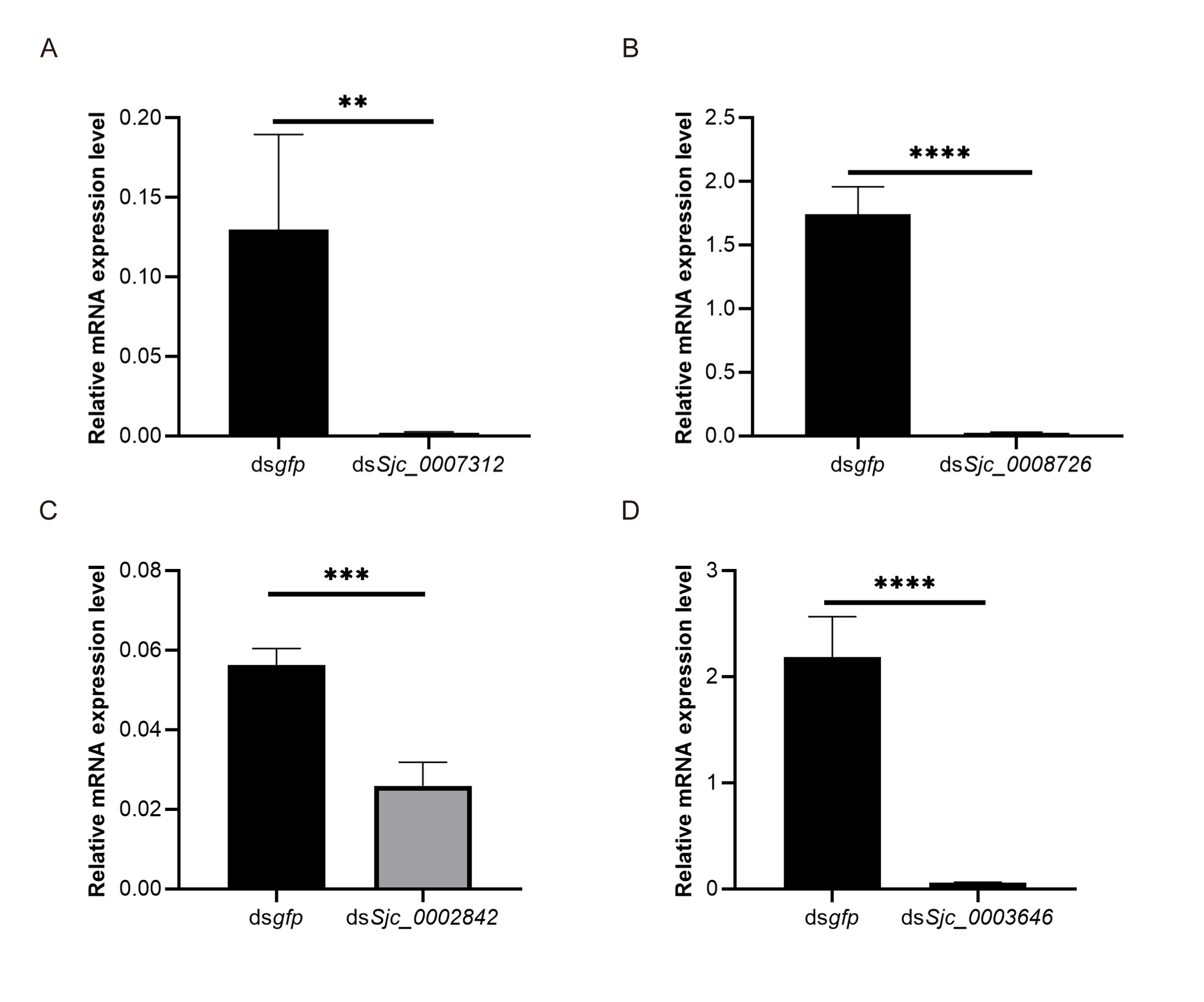

Supplement: S5 Fig — (A-D) Relative expression levels of Sjc_0007312 (A), Sjc_0008726 (B), Sjc_0002842 (C) and Sjc_0003646 (D) after dsRNA treatment for 10 days in males. Sjpsmd4 was used as the internal reference. Three biological replicates were performed. Error bars represent 95% confidence intervals. Differences are statistically significant (****p < 0.0001, ***p < 0.001, **p < 0.01, t-test). (TIF) [file ppat.1013014.s005.tif]

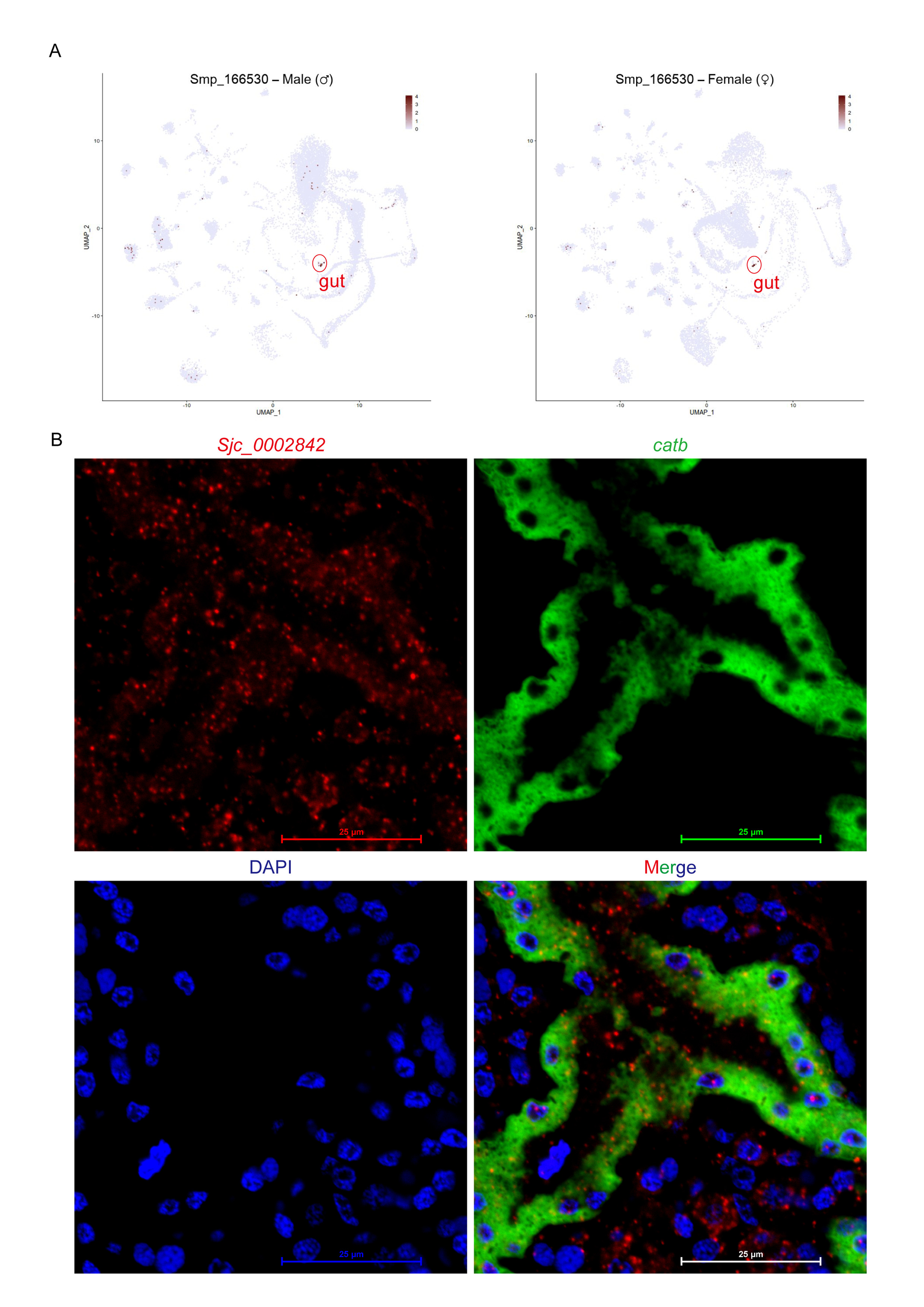

Supplement: S6 Fig — (A) UMAP projections depicting the expression profiles of Smp_166530 (homologous to Sjc_0002842 in S. japonicum) in different cell clusters in adult male (left) and female (right) S. mansoni. (B) Double FISH of Sjc_0002842 with the intestinal marker catb. Scale bars: 25 μm. (TIF) [file ppat.1013014.s006.tif]

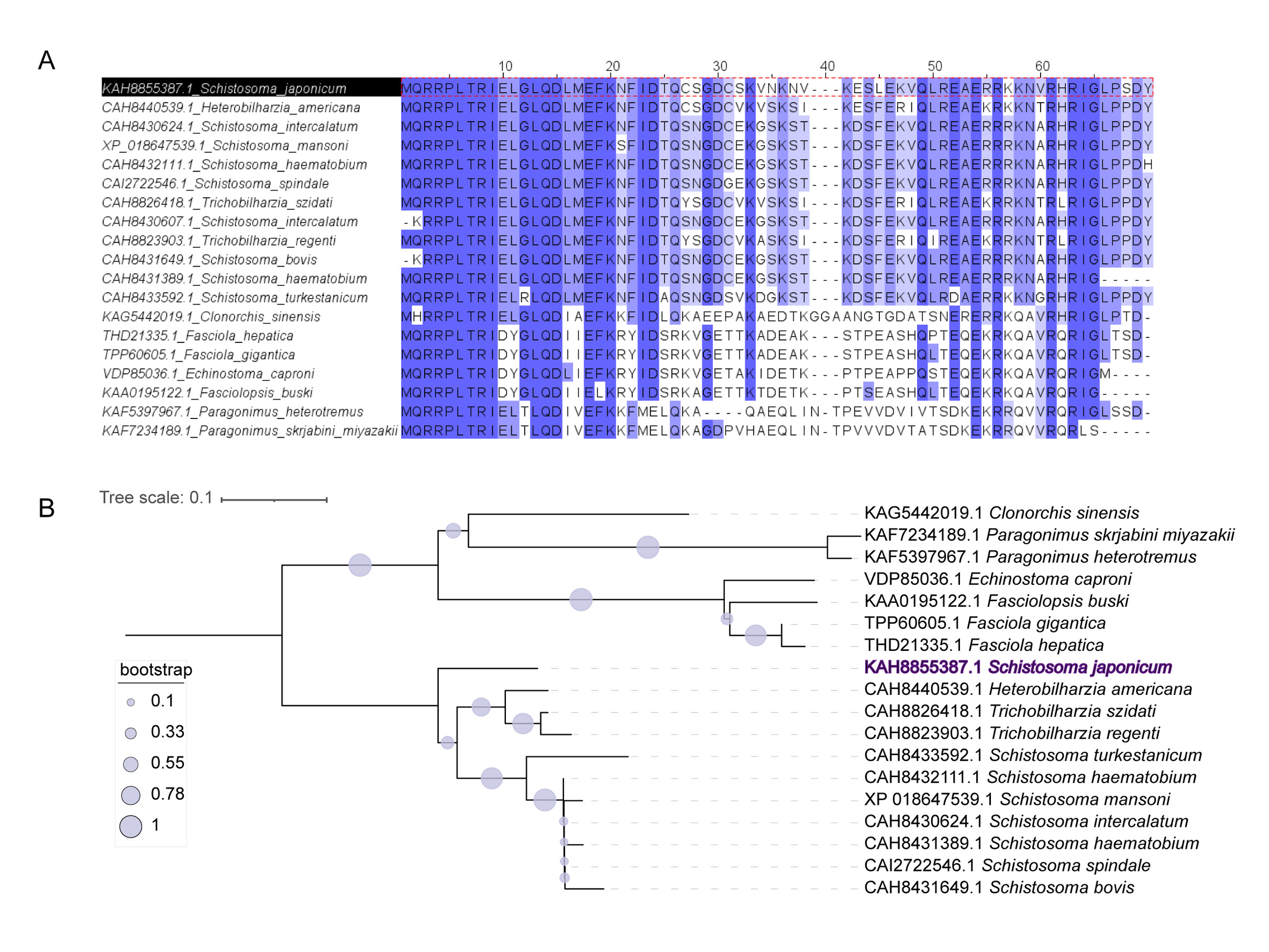

Supplement: S7 Fig — (A) Sequence alignment of Sjc_0002003 from S. japonicum with homologs from other species. The sequence ID includes the Genbank no. The Genbank no. displayed in white characters on a black background is Sjc_0002003. (B) Phylogenetic analysis of Sjc_0002003 and its homologs. The sequence ID includes the Genbank no.+ and species name. Sjc_0002003 is labelled in purple. (TIF) [file ppat.1013014.s007.tif]

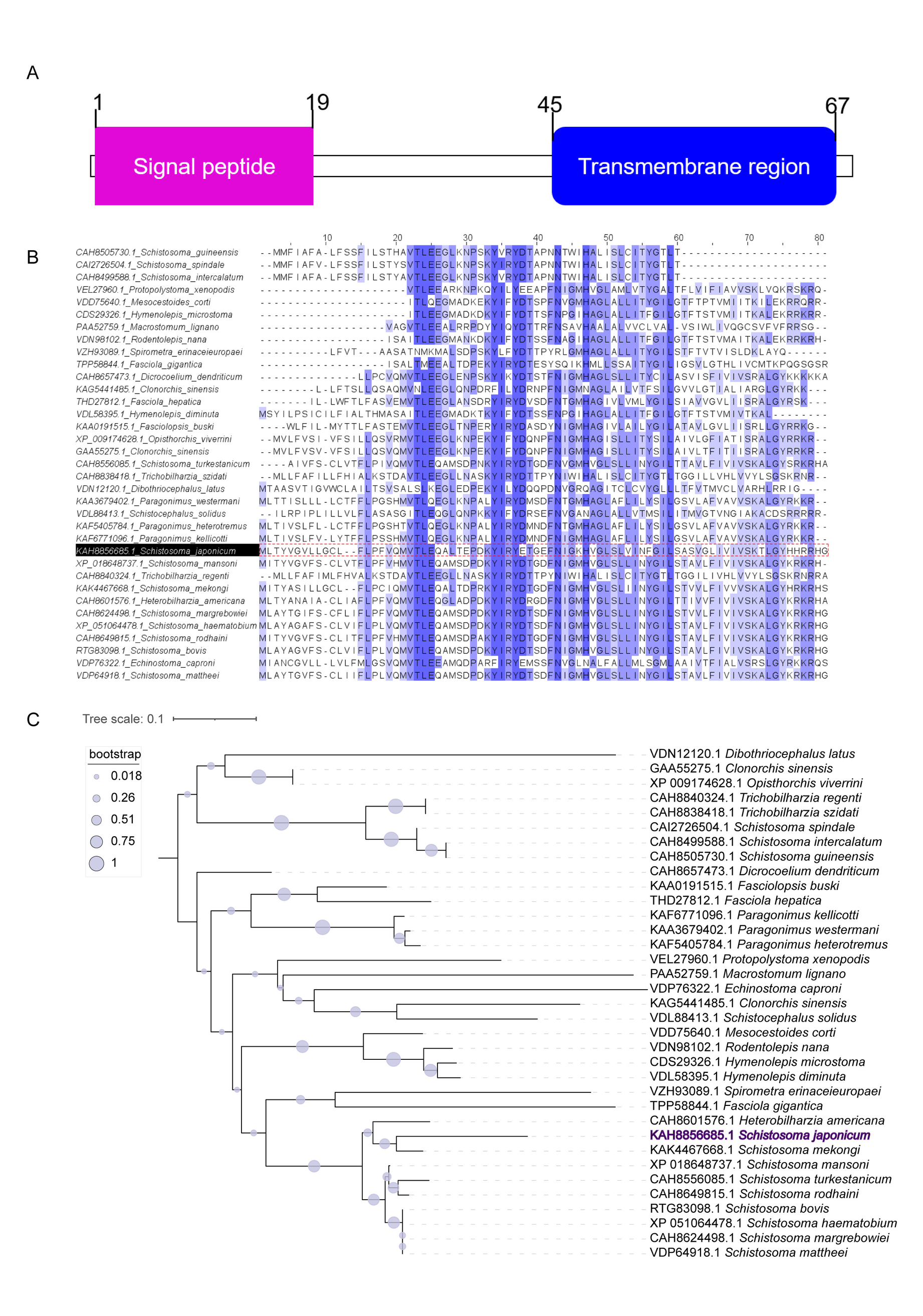

Supplement: S8 Fig — (A) Protein domains of Sjc_0009272. (B) Sequence alignment of Sjc_0009272 from S. japonicum with homologs from other species. The sequence ID includes the Genbank no. The Genbank no. displayled in white characters on a black background is Sjc_0009272. (C) Phylogenetic analysis of Sjc_0009272 and its homologs. The sequence ID includes the Genbank no. and species name. Sjc_0009272 is labelled in purple. (TIF) [file ppat.1013014.s008.tif]

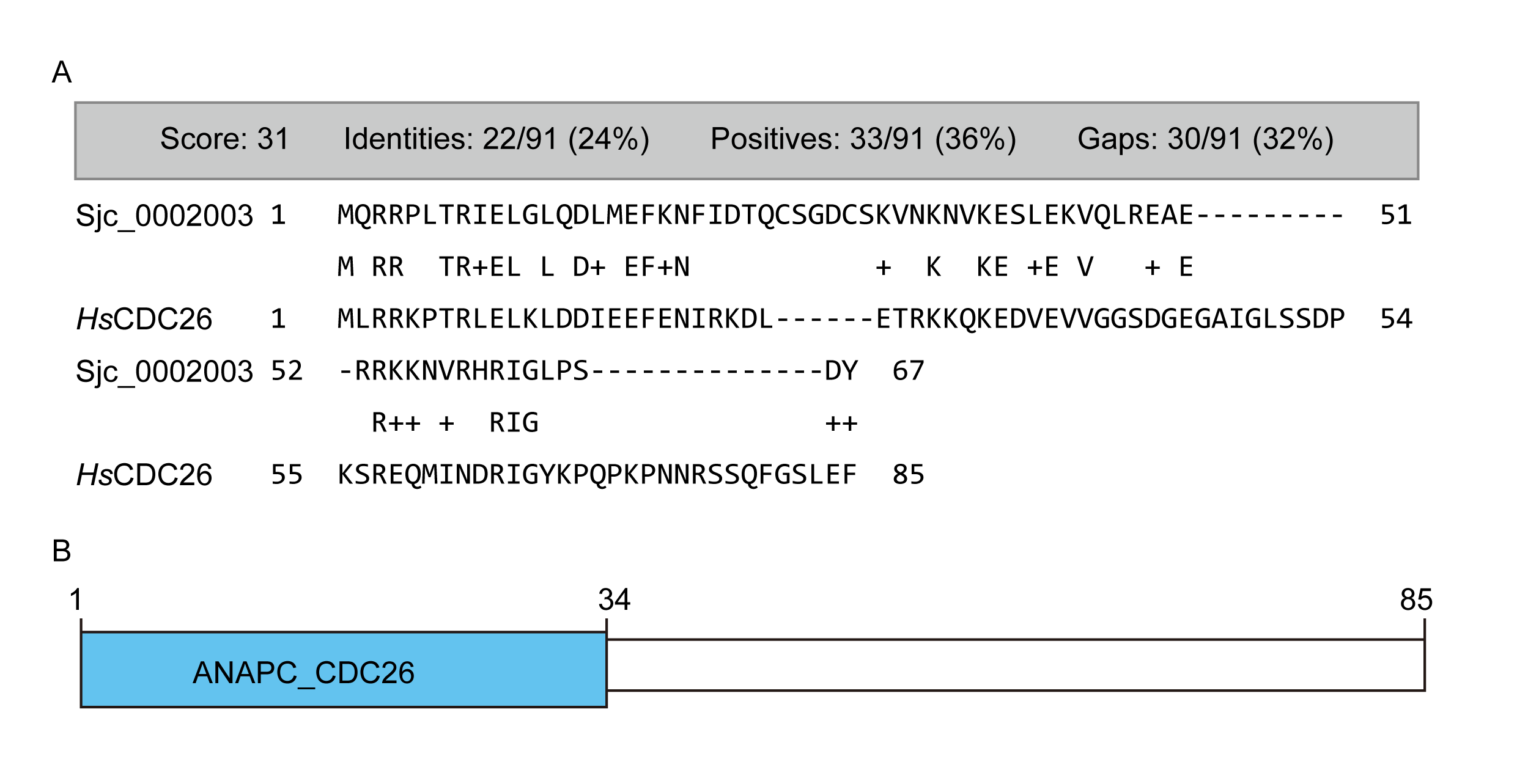

Supplement: S9 Fig — A) Needleman-Wunsch alignment of Sjc_0002003 and HsCDC26. (B) Identification of conserved domains on HsCDC26. (TIF) [file ppat.1013014.s009.tif]

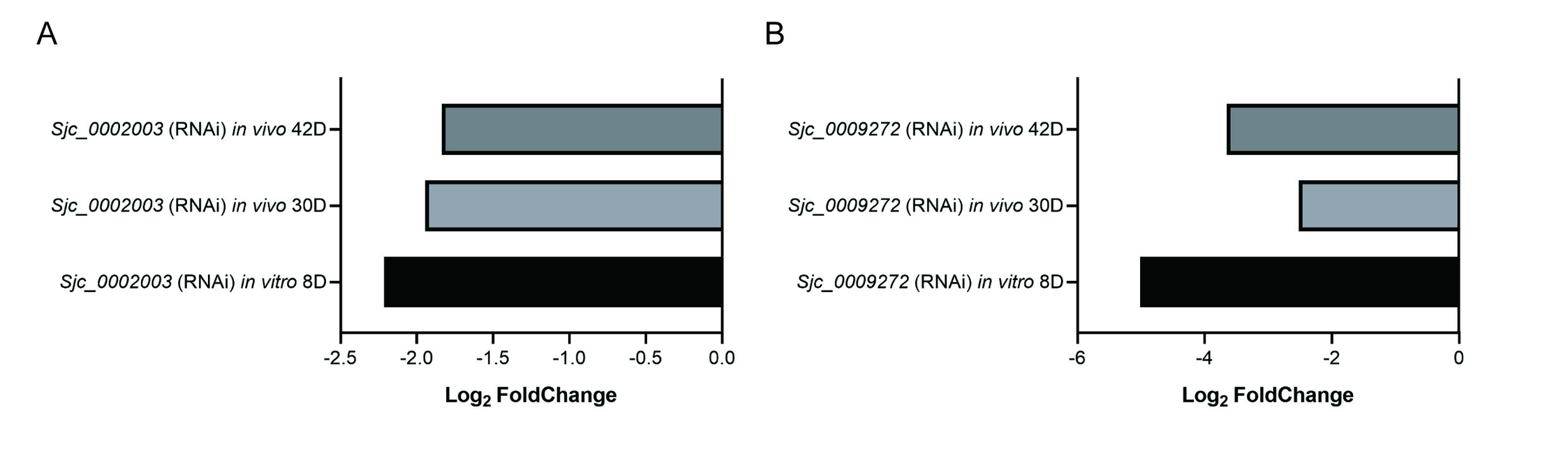

Supplement: S10 Fig — (A-B) Following dsRNA treatment and RNA-seq analysis, males showed a Log2 fold change in Sjc_0002003 (A) and Sjc_0009272 (B). In vitro, 8D worms were obtained after 8 days of dsRNA treatment. At 30 dpi, 30D worms were harvested in vivo from mice that had been injected via the tail vein with 10 μg of dsRNA targeting the specified genes on 1, 6, 10, 14, 18, 22, and 26 dpi. At 42 dpi, 42D worms were harvested in vivo from mice injected via the tail vein with 10 μg of dsRNA targeting the specified genes on 26, 30, 34, and 38 dpi. (TIF) [file ppat.1013014.s010.tif]

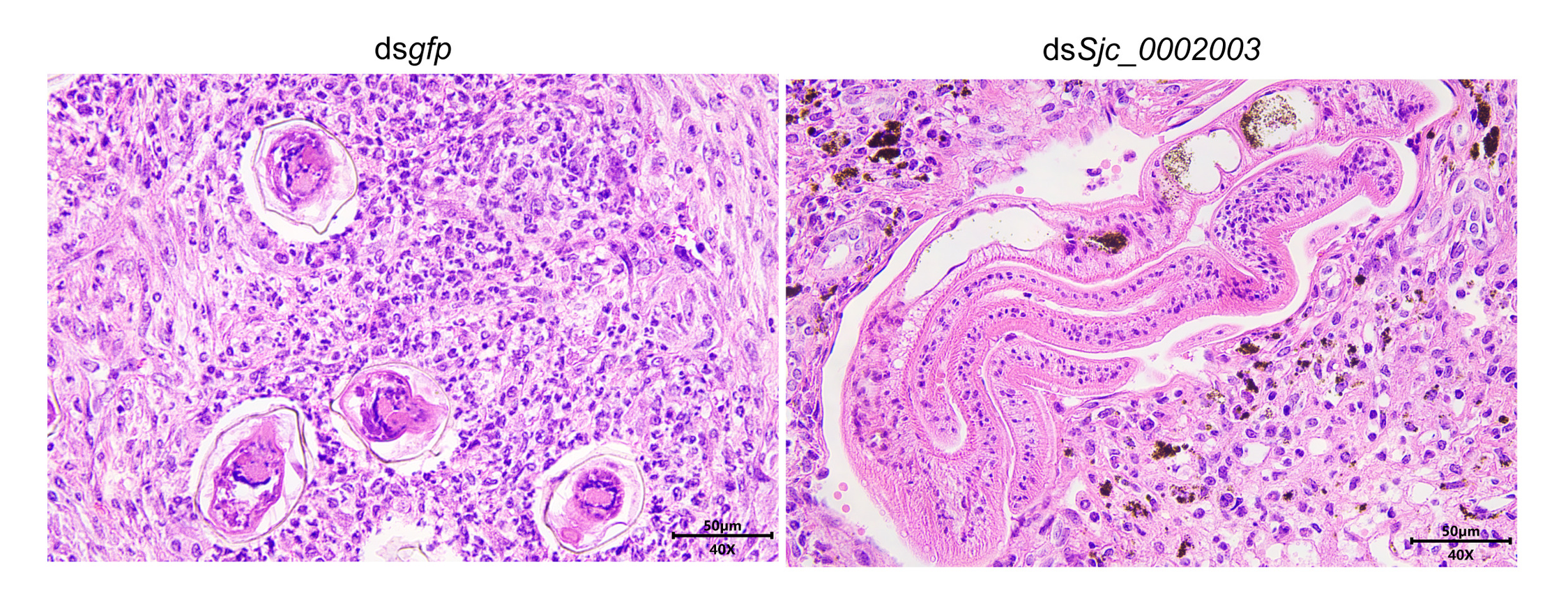

Supplement: S11 Fig — In the Sjc_0002003(RNAi) group, the worms deposited in the liver was surrounded by neutrophils and lymphocytes. Scale bars: 50 μm. (TIF) [file ppat.1013014.s011.tif]
